# Performance Analysis of Cluster Formation in Wireless Sensor Networks

**DOI:** 10.3390/s17122902

**Published:** 2017-12-13

**Authors:** Edgar Romo Montiel, Mario E. Rivero-Angeles, Gerardo Rubino, Heron Molina-Lozano, Rolando Menchaca-Mendez, Ricardo Menchaca-Mendez

**Affiliations:** 1 Instituto Politécnico Nacional—(CIC-IPN), Mexico City 07738, Mexico; eromom0900@alumno.ipn.mx (E.R.M.); hmolina@cic.ipn.mx (H.M.-L.); rmen@cic.ipn.mx (R.M.-M.); ric@cic.ipn.mx (R.M.-M.); 2 INRIA Rennes—Bretagne Atlantique, Campus Universitaire de Beaulieu, 35042 Rennes CEDEX, France; Gerardo.Rubino@inria.fr

**Keywords:** transmission probability, clustering, fuzzy C-means, K-medoids

## Abstract

Clustered-based wireless sensor networks have been extensively used in the literature in order to achieve considerable energy consumption reductions. However, two aspects of such systems have been largely overlooked. Namely, the transmission probability used during the cluster formation phase and the way in which cluster heads are selected. Both of these issues have an important impact on the performance of the system. For the former, it is common to consider that sensor nodes in a clustered-based Wireless Sensor Network (WSN) use a fixed transmission probability to send control data in order to build the clusters. However, due to the highly variable conditions experienced by these networks, a fixed transmission probability may lead to extra energy consumption. In view of this, three different transmission probability strategies are studied: optimal, fixed and adaptive. In this context, we also investigate cluster head selection schemes, specifically, we consider two intelligent schemes based on the fuzzy C-means and k-medoids algorithms and a random selection with no intelligence. We show that the use of intelligent schemes greatly improves the performance of the system, but their use entails higher complexity and selection delay. The main performance metrics considered in this work are energy consumption, successful transmission probability and cluster formation latency. As an additional feature of this work, we study the effect of errors in the wireless channel and the impact on the performance of the system under the different transmission probability schemes.

## 1. Introduction

Wireless Sensor Networks (WSNs) are deployed over a target area to supervise certain phenomena of interest. Each node takes readings from the local environment, processes and transmits a certain number of packets, containing the sensed data, to the sink node. Two common modalities can be used to access the shared medium to communicate the data to the sink node: unscheduled and scheduled-based transmissions [1,2]. In this paper, a clustered-based architecture is considered partly based on the encouraging results presented in previous works, such as [3,4]. In a cluster-based architecture, there are two distinct phases:

(1) The cluster formation phase, where all the active nodes transmit a control packet directed to the sink node in order to be part of the cluster. Specifically, the active nodes in the supervised area transmit their control packet with probability τ in each time slot. If there is only one transmission, that is only one node transmits, the control packet is successfully received by the sink node, and the node that successfully transmitted this packet is considered to be already a member of a cluster. As such, this node no longer transmits in the cluster formation phase. The remaining nodes continue this process until all the active nodes successfully transmit their control packet. If there are two or more transmissions in the same time slot, all transmissions are considered to be corrupted, and the control packets involved in this collision have to be retransmitted in future time slots. Hence, when a collision occurs, none of the involved nodes are aggregated to a cluster.
(2) The steady state phase, where all the nodes in the system transmit their data packets to a cluster head (CH), which in turn transmits an aggregated data packet to the sink node.

In the cluster formation phase, the active nodes transmit using a random access protocol where the channel is shared among all nodes, and hence, as stated before, collisions are possible. In this work, the slotted Non-Persistent Carrier-Sense Multiple Access (NP-CSMA) scheme is considered due to its superior performance compared to other variations of the Carrier-Sense Multiple Access (CSMA) protocol for WSN applications [3]. On the other hand, in the steady state, CHs assign resources by clarifying which sensor nodes should utilize the channel at any time through a Time Division Multiple Access (TDMA) protocol, thus ensuring a collision-free access to the shared data channel. One important characteristic of this phase is that only the transmitting nodes and their respective CHs are awake while the rest of the nodes go into sleep mode in order to save energy. For the sake of clarity, the packets used to form the clusters are referred to as control packets, while the packets used in the TDMA scheme will be referred to as data packets. Additionally, an adequate selection of the CH nodes can greatly reduce energy consumption. Indeed, a CH should be selected in order to reduce the distance to its cluster members (CMs) in order to also reduce the power transmission required to relay their information in the steady state phase. Therefore, intelligent clustering algorithms can reduce energy consumption by choosing the most appropriate nodes to become CHs or by reducing the average distance among the CMs and their respective CHs. As is shown in this paper, intelligent schemes are based on performing a number of iterations in order to find the most suitable CHs. This procedure also consumes time and energy. As such, in this work, we are interested in clearly identifying the conditions where the use of intelligent CH selection schemes greatly outperforms conventional CH selection schemes, such as the Low-Energy Adaptive Clustering Hierarchy (LEACH) protocol [5].

One main contribution of this work is to provide general guidelines on the selection of the transmission probability in the cluster formation phase on clustered WSNs. This issue has been largely neglected in the literature [6,7]. For simplicity, most previous work considered a fixed value of the transmission probability, which is selected independently of the network density [5,6,7,8,9,10,11]. This entails considerable energy wastage as is shown in the following sections. Previous work on WSNs attempt to reduce the collision probability by reducing the number of active nodes. For example, in [12], the authors propose the Correlation-based Collaborative Medium Access Control (CC-MAC) protocol that takes advantage of the spatial correlation inherent in such applications, in order to reduce the number of messages that have to be transmitted. Another approach proposed in [13] is to use multiple paths in order to reduce the collision probability. Yet another recent approach for reducing energy consumption in WSNs aims at using game theory to achieve an adequate performance, such as the work reported in [14,15,16]. Finally, in our previous work [17], we proposed different transmission probability selection schemes. However, neither errors in the channel were considered nor the CH selection procedure was presented. Furthermore, a new mathematical model was developed in this paper, which allowed us to find closed-form expression for the cluster formation delay and energy consumption, which were not derived in [17]. However, none of these works propose a suitable value of the transmission probability of the messages. As such, three different strategies for selecting the transmission probability in the cluster formation phase are studied here:

- Optimal transmission probability: For this strategy, the transmission probability that maximizes the successful transmission probability is used. The successful transmission probability is the probability that a node transmits a packet and it does not suffer a collision. This requires that all nodes in the surveilled area must be aware of the number of nodes that remain to transmit their control packet. In other words, all nodes in the system have to know the exact number of nodes that can potentially transmit in the next time slot. In a practical system, this is not feasible because there is no simple way to know the exact number of nodes inside the surveilled area since it is usually not fixed. Moreover, in many cases, the nodes are randomly deployed throughout the network. However, one way to implement this practically is to estimate the number of nodes inside the system by any means, if this is possible. Furthermore, observe that evaluating this optimal transmission strategy is of evident theoretical interest.
- Fixed transmission probability: In this scheme, a suitable value for the transmission probability is selected and remains unchanged during the cluster formation phase. As opposed to the optimal strategy, this scheme is very simple and easy to implement in a practical system. However, the selection of the value of the transmission probability is not straightforward, and it has a major impact on the performance of the system. This is because for high node densities, the transmission probability should be small in order to avoid a high number of collisions, and for low densities, the transmission probability should be high in order to avoid long idle-listening periods (that is, periods where there are no transmissions and where nodes have to continually listen to the channel). As such, once the transmission probability is appropriately selected for some particular conditions, the fixed transmission probability has a fair performance. The main problem with this strategy is that in WSNs, the system’s conditions are highly variable due to the death of nodes (nodes that consume all their battery’s power or are destroyed during the normal system operation) and to the addition of new nodes to the system. As such, when the number of nodes in the network changes, the performance of the system is degraded.
- Adaptive strategy: In this scheme, the transmission probability is adjusted according to the outcome of the previous slot. Specifically, the transmission probability is increased in the case of finding the channel idle; it is decremented in the case of collision; and it remains without change in the case of a successful transmission. In order to simplify the procedure and its tuning, the increment and decrement of the transmission probability is done according to a factor γ that has to be carefully selected. The performances achieved by this strategy are pretty close to those of the optimal one. It also has the advantage of constantly adapting to the conditions of the system. Hence, the death or addition of nodes has no important impact on the operation of the network. Finally, its practical implementation is easy since nodes only have to distinguish between a successful, collided or idle time slot, which is commonly used in previous work such as in [18]. It is important to notice that this scheme does not only adapt to different node densities, but it also adapts throughout the cluster formation procedure. Indeed, as the cluster begins to form, the initial number of nodes is relatively high, while at the end of the cluster formation phase, the number of nodes that can transmit is very low. Therefore, the transmission probability (τ) at the beginning of the cluster formation phase should be relatively small, while at the end of the cluster formation, this value should be close to one. This behavior is close to that of the optimal strategy, but with the advantage that there is no need to know the number of remaining nodes trying to transmit their control packets.

Since the adaptive and optimal schemes rely on the number of transmissions in the current time slot to calculate the value of the transmission probability, the effect of a noisy channel can drastically change the perception of the outcome in the current time slot. Indeed, based on the nature of the wireless communications, the presence of errors is unavoidable due to many factors like noise, interference and shadowing, among others. Building from this, we develop a Markov model to consider the effect of errors of the channel in the performance of the system.

Another important contribution of this work is the study and performance analysis of different CH selections in clustered-based WSNs. Specifically, we compare the performance of intelligent schemes where multiple iterations are performed in order to find the most appropriate nodes to act as CHs; and direct schemes where CHs are selected at random. Evidently, there are straightforward advantages of using intelligent schemes, such as a good CH distribution. However, there is an associated cost for their use. In this work, we aim at clearly identifying the scenarios where an intelligent scheme is strongly preferred in order to reduce energy consumption in the network and vice versa. It is important to note that we focus on the transmission probability selection in the cluster formation phase since in the steady state, transmissions are performed in an orderly fashion, where no collisions nor empty slots occur. As such, there is no room for improvement in this area in the steady state. However, this work also focuses on the performance in the steady state by means of the adequate cluster head selection algorithms. Indeed, the cluster head selection is performed at the cluster formation phase, but it has its impact on the steady state since it reduces long-range transmissions by evenly distributing cluster heads in the monitored area. Additionally, the cluster formation phase can be performed many times during the system operation. For instance, LEACH [5] proposes 20 s of steady state operation (called a round) and cluster formation after each round. The rationale behind this is that the node acting as the cluster head consumes much more energy since it is awake during the complete steady state operation gathering information from its cluster members and relaying this information to the sink node. Hence, every 20 s, a different node acts as the cluster head evenly distributing this high energy-consuming task. These 20 s rounds can be modified, but in general, it is expected that the round time will be in this range of a few seconds. Building on this, we consider that reducing energy consumption in the cluster formation is of major importance for the overall performance of the system.

In this work, we develop a simple Markov model to study the performance of the transmission strategies in the cluster formation phase in both ideal (error-free) and non-ideal channels (error-prone). The transmission strategies are studied in terms of energy consumption, successful transmission probability and cluster formation latency. Furthermore, two intelligent CH selection schemes are studied and analyzed: K-medoids [19] and fuzzy C-means [20]. These strategies are evaluated in terms of the number of iterations and average energy consumption. Furthermore, we compare these strategies to a single iteration CH selection strategy with no intelligence. The rest of the paper is organized as follows. Section 2 presents the mathematical model that describes the different transmission strategies used at the cluster formation phase in an error-free channel. Then, in Section 3, we develop the mathematical analysis to study the effect of errors in the wireless channels. Following this, Section 4 describes in detail the clustering schemes in the context of WSN. Section 5 presents a set of relevant numerical results. The article concludes with a summary of conclusions and contributions.

## 2. Model

In this section, the Markov chain used to model the random access protocol is described. We first present the main assumptions considered throughout the paper. Nodes are organized into clusters, each of which has a cluster head; the rest of the nodes become cluster members. We focus on the behavior of a given cluster. To form the clusters, all nodes transmit a packet directly to the sink node and continue to transmit that packet until it is successfully received. The sink node is situated outside the supervised area. All sensor nodes transmit with enough power to reach the sink node directly. A slotted NP-CSMA-based technique is used at the cluster formation and steady state phases. It is assumed that a packet can be transmitted in a single slot. Sensor nodes with a packet to transmit wait for the beginning of the next time slot and transmit with probability τ. Whenever a collision occurs, sensor nodes must retransmit their packet following the same procedure.

Whenever a node performs a transmission, it consumes $E_{t}$ units of energy, while for any reception, each node consumes $E_{r}$ units of energy. For the sake of clarity, it is considered that nodes only have a single transmission power level. In the numerical evaluations, we will set $E_{t}=1.0$ and $E_{r}=0.5$, but these values are studied in detail in Section 4. The case where the sensors run out of energy is not considered in this model, neither the case where new nodes are added to the system. However, in the Numerical Evaluation section, we present simulation results concerning the case where nodes deploy all their energy and new nodes are deployed in the system.

In the analysis, we naturally assume that the number of nodes inside the surveilled area is fixed and denoted by *N*. The transmission probability is defined as τ=P (sensor nodes use this probability to transmit their control packet in the cluster formation phase). Let us denote by $S_{h}$ the number of sensors that transmit when there are *h* active nodes. It follows that $S_{h}$ is a binomial random variable with parameters *h* and τ:

(1) P(Sh=j)=hjτj(1−τ)h−j

and $E(S_{h})=h\tau$.

For the fixed and optimal strategies, the aforementioned system can be modeled as a discrete-time Markov chain W={W(t),t∈N} where the states represent the number of nodes that have not yet successfully transmitted their packet. The state space of *W* is thus {N,N−1,⋯,1,0}, with W(0)=N. Denoting $p_{h}=P\left(S_{h}=1\right)=h\tau {\left(1-\tau \right)}^{h-1}$ by h≥1, the non-zero transition probabilities are:

(2) $$\begin{array}{c} P\left(W\left(t+1\right)=h-1|W\left(t\right)=h\right)=p_{h}, \end{array}$$

(3) $$\begin{array}{c} P\left(W\left(t+1\right)=h|W\left(t\right)=h\right)=1-p_{h}. \end{array}$$

**Figure 1:**
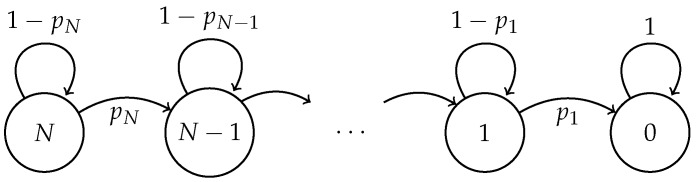
The Markov chain *W*.

Additionally, P(W(t+1)=0∣W(t)=0)=1 (that is, zero is an absorbing state). Figure 1 shows the graph of this Markov model.

For the adaptive scheme, a different Markov chain has to be considered since the value of τ is modified according to the outcome of the previous slot. The value of τ at the beginning of the cluster formation phase is set to some initial value $\tau_{0}$; it decreases after a collision by a factor γ>1, and it increases by the same factor γ if nobody attempted a transmission. We stop increasing this probability when it reaches the value $\tau_{0}\gamma^{\phi }$, and we stop decreasing it when its value is $\tau_{0}\gamma^{-\phi }$, for some positive integer ϕ. We denote $\tau_{j}=\tau_{0}\gamma^{j}$, j=−ϕ,−ϕ+1,…,ϕ. The state space of the chain is {N,N−1,…,1,0}×{−ϕ,−ϕ+1,…,ϕ}. State (n,j) means that there are *n* nodes attempting a transmission with transmission probability $\tau_{j}$ (we say “at phase *j*”).

**Figure 2:**
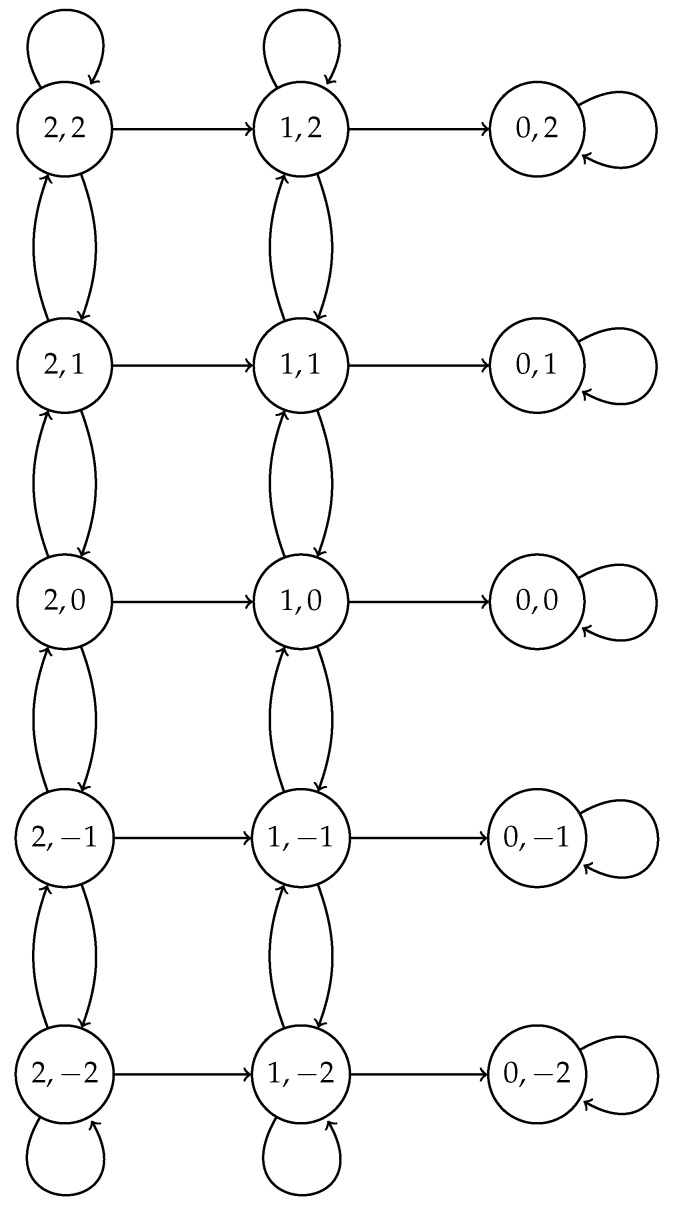
Markov chain for the adaptive case for N=2 nodes and ϕ=2. The transition probabilities are the $P_{x,y}$ given in the text.

States (0,ϕ),(0,ϕ−1),…,(0,−ϕ) are absorbing states; the remaining states are transient; the initial state is (N,0). In Figure 2, we show the valid state space and its corresponding transitions. Transition probabilities are as follows. Assuming the model is in state (n,j), n≥1. In the case of success, the next state is (n−1,j), which happens with probability $n\tau_{j}{\left(1-\tau_{j}\right)}^{n-1}$; if there are no transmissions, the next state is (n,j+1), with a higher transmission probability, which happens with probability ${\left(1-\tau_{j}\right)}^{n}$; otherwise, the next state is (n,j−1). If the phase is ϕ, the model stays at the same state if nobody transmits, that is, with probability ${\left(1-\tau_{\phi }\right)}^{n}$, goes to (n−1,ϕ) if there is a success (probability $n\tau_{\phi }{\left(1-\tau_{\phi }\right)}^{n-1}$) and goes to (n,ϕ−1) otherwise. If the phase is −ϕ, the next state is state (n−1,−ϕ) with probability $n\tau_{-\phi }{\left(1-\tau_{-\phi }\right)}^{n-1}$ and state (n,−ϕ+1) with probability ${\left(1-\tau_{-\phi }\right)}^{n}$, and the system remains at the same state otherwise. Formally, using the notation:

$$q_{n,j}=n\tau_{j}{\left(1-\tau_{j}\right)}^{n},\;r_{n,j}={\left(1-\tau_{j}\right)}^{n},$$

and $P_{x,y}$ for the transition probability from state *x* to state *y*, we have, for n≥1 and j≠−ϕ,ϕ:

$$\begin{array}{cc} P_{\left(n,j),(n-1,j\right)} & =q_{n,j},\\ P_{\left(n,j),(n,j+1\right)} & =r_{n,j},\\ P_{\left(n,j),(n,j-1\right)} & =1-q_{n,j}-r_{n,j}. \end{array}$$

At the borders, for n≥1,

$$\begin{array}{cc} P_{\left(n,\phi ),(n-1,\phi \right)} & =q_{n,\phi },\\ P_{\left(n,\phi ),(n,\phi \right)} & =r_{n,\phi },\\ P_{\left(n,\phi ),(n,\phi -1\right)} & =1-q_{n,\phi }-r_{n,\phi }, \end{array}$$

and:

$$\begin{array}{cc} P_{\left(n,-\phi ),(n-1,-\phi \right)} & =q_{n,-\phi },\\ P_{\left(n,-\phi ),(n,-\phi \right)} & =1-q_{n,-\phi }-r_{n,-\phi },\\ P_{\left(n,-\phi ),(n,-\phi +1\right)} & =r_{n,-\phi }. \end{array}$$

In the following sections, the average energy consumption and average cluster formation delay are derived for each transmission strategy.

### 2.1. Fixed Transmission Probability Scheme

The time that the Markov chain spends in state *h*, $T_{h}$, is geometrically distributed: for any h,m≥1,

(4) $$P\left(T_{h}=m\right)={\left(1-p_{h}\right)}^{m-1}p_{h}.$$

The expected time that the system remains in state *h* is thus:

(5) $$E\left(T_{h}\right)=1/p_{h}={\left[h\tau {\left(1-\tau \right)}^{h-1}\right]}^{-1}.$$

Therefore, the expected time to form the cluster is:

(6) $$E\left(T_{\mathrm{fixed}}\right)=\sum_{h=1}^{N}{\left[h\tau {\left(1-\tau \right)}^{h-1}\right]}^{-1}.$$

The variance of the time spent in state *h* is:

(7) $$V\left(T_{h}\right)=\frac{1-p_{h}}{p_{h}^{2}}.$$

Hence, the variance of the cluster formation time is:

(8) $$V\left(T_{\mathrm{fixed}}\right)=\sum_{h=1}^{N}\frac{1-p_{h}}{p_{h}^{2}}.$$

Observe that the sum of geometrically-distributed random variables has a coefficient of variation less than one. This implies that the mean cluster formation time is quite stable in the sense that it does not have high variations.

In order to calculate the average energy consumption, observe that when the system is in state *h*, whenever a successful transmission occurs, there is one node that consumes $E_{t}$ units of energy, while there are h−1 nodes that receive the packet, each consuming $E_{r}$ units of energy. Hence, the energy consumption in the case of a successful transmission is $E_{t}+\left(h-1\right)E_{r}$. On the other hand, whenever a collision occurs or if there are no transmissions, there are $S_{h}$ nodes that transmit (h≠1), each one consuming $E_{t}$ units of energy while $h-S_{h}$ nodes listen to the channel consuming $E_{r}$ units of energy each.

Let $C_{h}$ denote the energy consumption during the period where there are *h* sensors left at the cluster formation phase. Recall that $T_{h}$ is the number of steps to go from *h* to h−1, that is, the number of steps until a success. Let us compute the average energy consumption in this state *h*. We start by conditioning on the length of the sojourn in state *h* to get:

(9) $$E\left(C_{h}\right)=\sum_{k=1}^{\infty }E\left[C_{h}|T_{h}=k\right]P\left(T_{h}=k\right).$$

On average, there will be 1/$p_{h}-1$ unsuccessful time slots and one successful transmission. Denoting by $\alpha_{h}$ the mean cost of an unsuccessful slot in state *h*, we have:

(10) $$E\left[C_{h}|T_{h}=m\right]=\left(m-1\right)\alpha_{h}+E_{t}+\left(h-1\right)E_{r}.$$

Substituting this in Equation (9), we get:

(11) $$E\left[C_{h}\right]=\alpha_{h}\frac{1-p_{h}}{p_{h}}+E_{t}+\left(h-1\right)E_{r}.$$

Now, $\alpha_{h}$ can be obtained as follows:

$$\begin{array}{cc} \alpha_{h} & =\sum_{j=0}^{h}P\left(S_{h}=j|S_{h}\neq 1\right)\left[jE_{t}+\left(h-j\right)E_{r}\right]\\ & =\sum_{j=0,j\neq 1}^{h}\frac{P(S_{h}=j)}{P(S_{h}\neq 1)}\left[\left(E_{t}-E_{r}\right)j+hE_{r}\right]\\ & =\left(E_{t}-E_{r}\right)\frac{h\tau -p_{h}}{1-p_{h}}+hE_{r}. \end{array}$$

Substituting this expression in Equation (11), we get, after some algebra:

(12) $$E\left(C_{h}\right)=\frac{\tau \left(E_{t}-E_{r}\right)+E_{r}}{\tau {\left(1-\tau \right)}^{h-1}}.$$

Let us denote by $C_{\mathrm{fixed}}$ the total energy consumption per cluster formation. On average, we have:

(13) $$E\left(C_{\mathrm{fixed}}\right)=\sum_{h=1}^{N}E\left(C_{h}\right).$$

Using Equation (12), we finally obtain:

(14) $$E\left(C_{\mathrm{fixed}}\right)=\frac{\left(1-\tau \right)[\tau \left(E_{t}-E_{r}\right)+E_{r}]}{\tau^{2}}\left[{\left(\frac{1}{1-\tau }\right)}^{\;\;N}-1\right].$$

If τ is small, this can be approximated by:

$$E\left(C_{\mathrm{fixed}}\right)=\frac{NE_{r}}{\tau }+\frac{E_{r}N^{2}+\left(2E_{t}-3E_{r}\right)N}{2},$$

with the missing remaining term being o(τ).

By using the numerical values $E_{t}=1$, $E_{r}=1/2$, the expected cost is:

$$E\left(C_{\mathrm{fixed}}\right)=f_{N}\left(\tau \right)=\frac{1-\tau^{2}}{2\tau^{2}}\left[{\left(\frac{1}{1-\tau }\right)}^{\;\;N}-1\right].$$

We can show that $f_{N}$ has a single minimum in the interval [0,1], and that if we denote it by $\tau_{N}$, we have:

$$\frac{1}{N}<\tau_{N}<\frac{2}{N}.$$

To do this, we compute the first derivative:

$$f_{N}^{\prime }\left(\tau \right)=\frac{1}{\tau^{3}}+\frac{1}{\tau^{2}}{\left(\frac{1}{1-\tau }\right)}^{\;\;N}\;\;\;\left(N\tau^{2}+N\tau -2\right).$$

Solving $f_{N}^{\prime }\left(\tau \right)=0$ in [0,1] is equivalent to solving the equation $u_{N}\left(\tau \right)=v_{N}\left(\tau \right)$ in that interval, where $u_{N}\left(\tau \right)=2{\left(1-\tau \right)}^{N}$ and $v_{N}\left(\tau \right)=2-N\tau -N\tau^{2}$. The analysis of these two functions on [0,1] (and in particular, the observation that $u_{N}^{\prime }\left(0\right)=-2N$ and $v_{N}^{\prime }\left(0\right)=-N$) shows that there is a single minimum at a point $\tau_{N}\in \left(1/N,2/N\right)$. Since *N* is expected to be at least on the order of tens, this suggests that we can roughly give the following approximate value:

(15) $$\tau_{N}\approx \frac{3}{2N}.$$

This gives an optimal expected cost slightly less than $N^{2}$, as a first approximation. This value is obtained by replacing τ by the approximating value of 3/(2N) in $f_{N}$, and by looking at an equivalence when N→∞, we have:

$$f_{N}\left(\frac{3}{2N}\right)=\frac{1}{2}\left(\frac{4}{9}N^{2}-1\right)\left[{\left(1-\frac{3}{2N}\right)}^{-N}-1\right],$$

and then, we can use: ${\left(1-3/(2N)\right)}^{-N}\to e^{3/2}$. to get:

$$f_{N}\left(\frac{3}{2N}\right)∼\frac{2}{9}\left(e^{3/2}-1\right)N^{2}\approx 0.7737N^{2}.$$

### 2.2. Optimal Transmission Probability Scheme

This scheme is considered to be optimal in the sense that it maximizes the probability of having a successful transmission. We simply solve $dp_{h}/d\tau =0$ as an equation in τ. From $p_{h}\;=\;P\left(S_{h}\;=\;1\right)\;=\;h\;\tau {\left(1-\tau \right)}^{h-1}$, we get:

(16) $$\frac{dp_{h}}{d\tau }=h{\left(1-\tau \right)}^{h-1}-h\tau \left(h-1\right){\left(1-\tau \right)}^{h-2}=0,$$

and obtain that the best value for $p_{h}$ corresponds to $\tau =\tau_{h}^{*}=1/h$, and its value is:

$$p_{h}^{*}={\left(1-\frac{1}{h}\right)}^{h-1}.$$

Hence, when the system is in state *h*, all remaining nodes in the cluster formation process have to transmit with probability 1/h. From this, it is clear that in order to implement this scheme, the sensor nodes have to know the exact number of nodes that have not successfully transmitted their control packets. As such, the number of nodes in the network also needs to be known. This is the main problem of using this strategy.

For the time cluster formation, its expected value for the optimal strategy is:

(17) $$E\left(T_{\mathrm{opt}}\right)=1+\sum_{h=2}^{N}{\left(\frac{h}{h-1}\right)}^{\;\;h-1}$$

and the average energy consumption per cluster formation can be calculated as:

(18) $$E\left(C_{\mathrm{opt}}\right)=E_{t}+2E_{r}+\sum_{h=2}^{N}\left[\left(E_{t}+E_{r}\right)+hE_{r}\right]{\left(\frac{h}{h-1}\right)}^{h-1}.$$

### 2.3. Adaptive Transmission Probability Scheme

For the adaptive scheme, the initial value of the transmission probability is selected as τ=1/N. Note that unlike the optimum strategy, it is not necessary to know the exact value of *N* since the nodes constantly adapt the value of τ according to the outcome of the last slot as previously explained (more on this in the next section). First, the expected cluster formation latency is calculated. The expected number of time slots to reach the absorbent state (0, *m*) starting in state (*N*, 0) is given by:

(19) $$E\left(T_{\mathrm{adaptive}}\right)=v_{N,0},$$

where $v_{n,j}$ denotes the expected absorption time starting at the state (n,j) of the chain (so, in particular, $v_{0,j}=0$ for j=−ϕ,−ϕ+1,…,ϕ). These conditional expectations are computed by solving the following linear system:

(20) $$v_{n,j}=1+\sum_{h=0}^{N}\sum_{\ell =-\phi }^{\phi }P_{\left(n,j),(h,\ell \right)}v_{h,\ell },$$

where the $P_{\left(n,j),(h,\ell \right)}$s have been given when the Markov chain was described.

For the case of the energy consumption, $C_{ij,kl}$ denotes the energy cost associated with the transition from state (*i*,*j*) to state (*k*,*l*). Then, $C_{ij}=\sum_{k=1}^{N}\sum_{l=-\phi }^{\phi }P_{ij,kl}C_{ij,kl}$ is the expected energy consumption cost associated with a transition from state (*i*,*j*). As such, the energy consumption from the state (i,j) to the absorbent state (0,0) can be calculated as:

(21) $$EC_{i,j}=C_{ij}+\sum_{k=1}^{N}\sum_{l=-\phi }^{\phi }P_{ij,kl}EC_{kl}$$

where $C_{i,j}=i\tau_{j}{\left(1-\tau_{j}\right)}^{i-1}\left(1+\left(i-1\right)E_{rx}\right)+{\left(1-\tau_{j}\right)}^{i}\left(iE_{rx}\right)+\left[1-i\tau_{j}{\left(1-\tau_{j}\right)}^{i-1}-{\left(1-\tau_{j}\right)}^{i}\right]\left(iE_{rx}+\left(1-E_{rx}\right)i\tau_{j}\frac{1-{\left(1-\tau_{j}\right)}^{i-1}}{1-i\tau_{j}{\left(1-\tau_{j}\right)}^{i-1}}\right)$. Hence, the expected energy consumption at the cluster formation phase is given by:

(22) $$E\left(C_{\mathrm{adaptive}}\right)=EC_{N,0}$$

From the previous analysis, we show in the Numerical Evaluation section that the optimum scheme always achieves the lowest average energy consumption and average cluster formation delay. However, to achieve this, the scheme requires perfect knowledge of the cluster formation process, i.e., nodes have to know the exact number of remaining nodes to transmit their control packet, as seen in Equation (17), where the transmission probability is 1/h (where *h* is the number of active nodes.) This is not always possible, as shown in the following section where a noisy channel affects the exact knowledge of active nodes or even because nodes do not have such capabilities due to hardware restrictions. Conversely, the fixed scheme does not have these drawbacks since a single probability is used throughout the cluster formation phase. Hence, no node estimation is required. However, it achieves the highest average consumption and average cluster formation delay, even if the optimum transmission probability, shown in Equation (15), is used. The adaptive scheme achieves very close results compared to the optimal scheme without the need for knowing the exact number of active nodes assuming a good selection of the value of γ. This issue is explored in detail in the following sections.

## 3. Model Considering a Noisy Channel

In this section, we present a model considering the presence of errors in the communication channel. Errors in the packet reception can occur due to interference, shadowing, multiple trajectories of the signal and noise, among others. In this work, we consider that due to these errors, the receiving nodes can infer that a transmission did not occur, or a single transmission can be perceived as a collision, or even that a transmission happened when no node transmitted in the time slot. In view of this, we consider the use of the following probabilities associated with the effect of errors in the wireless channel as presented in [21,22,23,24]:

- False positive is an event in which no node transmits its data packet in the current slot, but the nodes detect that one successful transmission has occurred. This occurs with probability $P_{e}^{+}$.
- False negative is an event in which only one node transmits, but it is not successfully decoded at the receiver. This event is assumed to occur with probability $P_{e}^{-}$.

With this model for errors, we intend to consider different phenomena that occur in the wireless channel, such as multipath fading, noise, shadowing and obstacles in the trajectory, among others. Now, we investigate the effect of these errors in the previously-explained transmission probability strategies.

### 3.1. Fixed Transmission Probability Scheme with Channel Errors

Since nodes transmit with a fixed probability, a successful transmission occurs when one of the following cases occurs:

- Only one node transmits and the channel is error-free, i.e., neither a false positive nor a false negative probability happens during the transmission.
- We assume that the channel is error-free when both false positive and false negative events occur in the same slot, because these events are mutually exclusive.

The former case occurs with probability $\left(1-P_{e}^{+}\right)\left(1-P_{e}^{-}\right)$, and the latter case occurs with probability $P_{e}^{+}P_{e}^{-}$.

**Figure 3:**
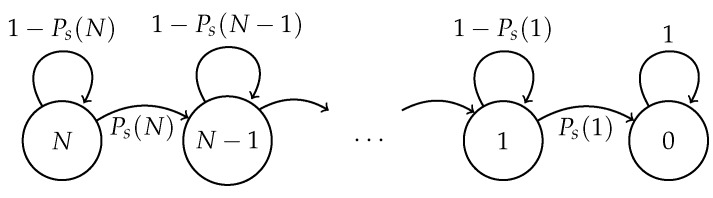
Markov chain for the cluster formation phase with fixed transmission probability and channel errors.

Building on this, the Markov chain that describes the cluster formation phase is depicted in Figure 3, where:

$$\begin{array}{cc} P_{s}\left(N\right) & =P\left(S_{h}=1\right)\left(1-P_{e}^{+}\right)\left(1-P_{e}^{-}\right)+P\left(S_{h}=1\right)P_{e}^{+}P_{e}^{-}\\ & =P\left(S_{h}=1\right)\left[\left(1-P_{e}^{+}\right)\left(1-P_{e}^{-}\right)+P_{e}^{+}P_{e}^{-}\right]\\ & =P\left(S_{h}=1\right)\left[1-P_{e}^{-}\left(1-P_{e}^{+}\right)-P_{e}^{+}\left(1-P_{e}^{-}\right)\right] \end{array}$$

### 3.2. Optimal Transmission Probability Scheme with Channel Errors

Recall that in this scheme, nodes attempting to transmit adjust their value of τ according to the number of remaining active nodes in the cluster formation phase. As such, the occurrence of errors has a major impact on the performance of the system as explained now in detail:

- In the presence of false positives, active nodes estimate that there is one less node in the cluster formation procedure even if no node transmitted. Hence, the remaining nodes increase the value of τ. However, the actual number of nodes attempting to transmit remains unchanged; consequently, a non-optimal value of τ is now being used during the remaining procedure.
- When only one node transmits and a false positive occurs, a collision is detected, then that node has to retransmit in some future time slot. The impact in the system is similar to the previous case.
- On the other hand, false negative cases do not greatly affect the system’s performance. Indeed, in this case, the system fails to detect a successful transmission. Hence, the value of τ is not modified, and nodes continue to use an adequate value of the transmission probability.

**Figure 4:**
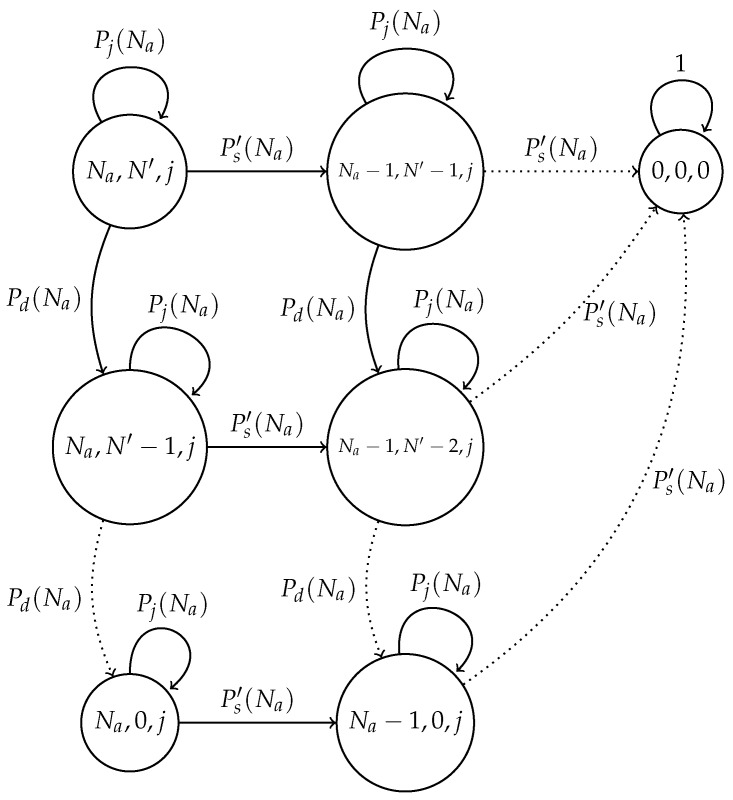
Markov chain for the optimal transmission probability scheme in an error-prone channel. $P_{s}^{\prime }$ represents the successful transmission probability; $P_{j}$ is the probability that *j* nodes transmit, but the chain will remain in the same state; and $P_{d}$ is the probability that the network estimates less nodes attempting to transmit than the actual value of nodes.

For this error-prone system, we developed a Markov chain model in which each state represents the actual number of nodes attempting to transmit; the estimated number of nodes attempting to transmit and the number of nodes that transmit in each time slot are denoted by ($N_{a},N^{\prime },j$), respectively, with $N_{a},N^{\prime }\geq 0$ and $0\leq j\leq N_{a}$. State (0,x,0), x≥0 is an absorbing state. Figure 4 shows this model.

We now detail the transition probabilities of the proposed chain. First, the probability of a successful transmission is given by $P_{s}^{\prime }\left(N_{a}\right)$, which depicts the case where only one node transmits in the current slot and the channel is considered as error-free. This probability is given by:

$$\begin{array}{cc} P_{s}^{\prime }\left(N_{a}\right) & =P\left(S_{h}=1\right)\left(1-P_{e}^{+}\right)\left(1-P_{e}^{-}\right)+P\left(S_{h}=1\right)P_{e}^{+}P_{e}^{-}\\ & =P\left(S_{h}=1\right)\left[\left(1-P_{e}^{+}\right)\left(1-P_{e}^{-}\right)+P_{e}^{+}P_{e}^{-}\right]\\ & =P\left(S_{h}=1\right)\left[1-P_{e}^{-}\left(1-P_{e}^{+}\right)-P_{e}^{+}\left(1-P_{e}^{-}\right)\right] \end{array}$$

Note that we use the value of $P_{s}^{\prime }$ as opposed to $P_{s}$, since a different value of τ is used in the error-prone network. Specifically, in the error-free system, τ=1/N, while in the error-prone system, $\tau =1/N^{\prime }$. Furthermore, note that the values of *N* and $N^{\prime }$ can greatly differ from each other according to the channel conditions in terms of the values of the error probabilities.

Additionally, a special event occurs when no node transmits, but a false positive is detected in the system (consequently no false negatives occur). In this case, the nodes in the network listen for a successful transmission and estimate that there is one less node attempting to transmit in future slots. This occurs with probability $P_{d}\left(N_{a}\right)$, which is computed below.

$$\begin{array}{cc} P_{d}\left(N_{a}\right) & =P\left(S_{h}=0\right)P_{e}^{+}\left(1-P_{e}^{-}\right)\\ & ={\left(1-1/N^{\prime }\right)}^{N_{a}}P_{e}^{+}\left(1-P_{e}^{-}\right) \end{array}$$

Finally, a set of events is considered in the model through the transition labeled with the probability $P_{j}\left(N_{a}\right)$. These events do not change the state of the chain and involve two general cases: idle channel and collisions.

Idle slots occur when no node transmits and no errors occur or when only one node transmits, but errors in the channel corrupt such transmission. Collided slots occurs when only one node transmits and a false positive (and consequently no false negative) happens. Additionally, when two or more nodes transmit in the same slot, a collision occurs independently of the errors in the channel. Hence, $P_{j}\left(N_{a}\right)$ is computed as follows.

$$\begin{array}{cc} P_{j}\left(N_{a}\right) & =P\left(S_{h}=0\right)\left[P_{e}^{+}P_{e}^{-}+\left(1-P_{e}^{+}\right)\left(1-P_{e}^{-}\right)\right]\\ & +P\left(S_{h}=0\right)P_{e}^{-}\left(1-P_{e}^{+}\right)+P\left(S_{h}=1\right)P_{e}^{-}\left(1-P_{e}^{+}\right)\\ & +P\left(S_{h}=1\right)P_{e}^{+}\left(1-P_{e}^{-}\right)+\sum_{j\geq 2}^{N_{a}}P\left(S_{h}\right) \end{array}$$

### 3.3. Adaptive Transmission Probability Scheme with Channel Errors

**Figure 5:**
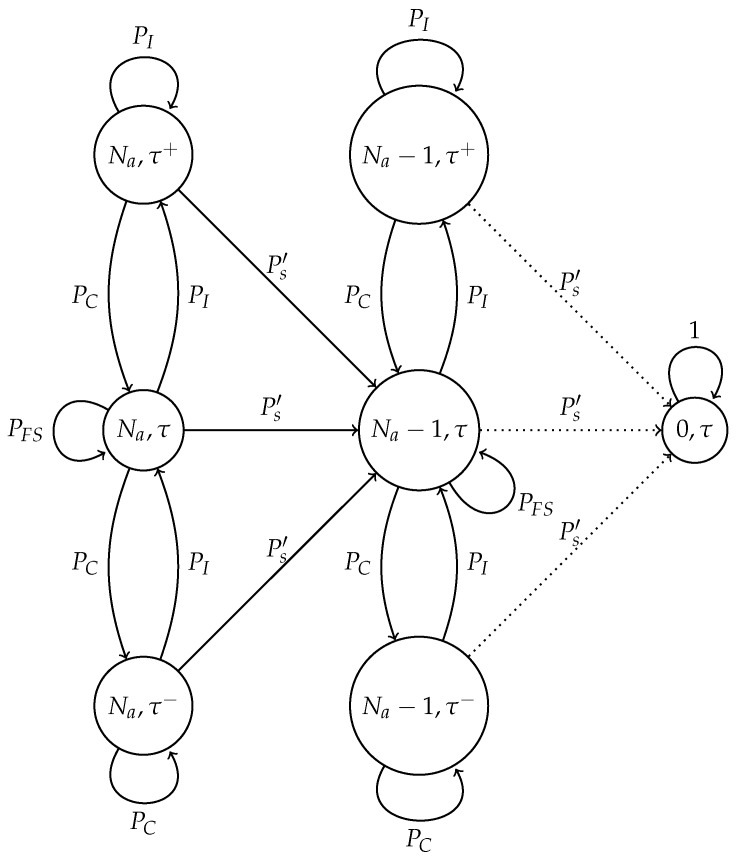
Markov chain for the adaptive scheme in an error-prone channel. $P_{s}^{\prime }$ represents the successful transmission probability; $P_{C}$ is the probability of collision; $P_{I}$ is the idle channel probability; and $P_{FS}$ is the probability of false success.

In this subsection, we present the model of the adaptive transmission probability scheme in an error-prone channel. In this case, four possible events are contemplated: idle channel, collision, successful transmission and false success. For this system, we developed the Markov chain presented in Figure 5.

The model is now described in detail. The state of the chain is given as ($N_{a},\tau$), where $N_{a}$ is the actual number of nodes in the system. As opposed to the optimal scheme, it is not necessary to know the value of an estimated number of nodes. Furthermore, recall that $\tau^{+}$ represents the case where the current value of τ increases while $\tau^{-}$ represents the case where τ decreases.

First, we analyze the transition labeled by $P_{s}^{\prime }$, which is the probability of a successful transmission. This probability is given, as in the optimal scheme, by:

$$\begin{array}{cc} P^{\prime }s & =P_{s}^{\prime }\left(N_{a}\right)=P\left(S_{h}=1\right)\left[\left(1-P_{e}^{+}\right)\left(1-P_{e}^{-}\right)+P_{e}^{+}P_{e}^{-}\right]\\ & =P\left(S_{h}=1\right)\left[1-P_{e}^{-}\left(1-P_{e}^{+}\right)-P_{e}^{+}\left(1-P_{e}^{-}\right)\right] \end{array}$$

A collision is presented in the system with probability $P_{C}$, which involves the events when one node transmits and a false positive occurs and when two or more nodes transmit in the same time slot. This probability can be expressed as follows:

$$\begin{array}{c} P_{C}=P\left(S_{h}=1\right)P_{e}^{+}\left(1-P_{e}^{-}\right)+\sum_{j\geq 2}^{N_{a}}P\left(S_{h}\right) \end{array}$$

When a collision occurs, the value of τ is updated as τ(t+1)=τ(t)γ; with *t* representing the current time slot.

On the other hand, an idle time slot occurs with probability $P_{I}$ and can occur when one of the following events occurs:

- No node transmits and the channel is error-free.
- No node transmits and a false negative occurs (consequently, no false positive occurs.)
- One node transmits and a false negative happens (consequently, no false positive occurs).

Building on this, an idle slot occurs with probability $P_{I}$, which is given by:

$$\begin{array}{cc} P_{I} & =P\left(S_{h}=0\right)\left[P_{e}^{+}P_{e}^{-}+\left(1-P_{e}^{+}\right)\left(1-P_{e}^{-}\right)\right]\\ & +P\left(S_{h}=0\right)P_{e}^{-}\left(1-P_{e}^{+}\right)+P\left(S_{h}=1\right)P_{e}^{-}\left(1-P_{e}^{+}\right)\\ P_{I} & =P\left(S_{h}=0\right)\left[1-P_{e}^{+}\left(1-P_{e}^{-}\right)\right]+P\left(S_{h}=1\right)P_{e}^{-}\left(1-P_{e}^{+}\right) \end{array}$$

In this case, the value of τ is updated as τ(t+1)=τ(t)1/γ. Finally, the probability of false success $P_{FS}$ is the event where a false positive occurs. Hence:

$$\begin{array}{c} P_{FS}=P\left(S_{h}=0\right)P_{e}^{+}\left(1-P_{e}^{-}\right) \end{array}$$

### 3.4. Threshold Selection

The optimal and adaptive transmission probability schemes offer a solution to reduce energy consumption and delay in the cluster formation process. However, in the presence of errors, the selection of τ is not straightforward as described as follows:

- In the optimal scheme, one particularly harmful event that can occur due to a noisy channel is when the estimated number of active nodes is low (i.e., the network estimates that most nodes have successfully transmitted their control packet), but in fact, there is a higher amount of nodes still active in the cluster formation phase. This situation can occur if the false positive probability is rather high. In this case, the estimated value of τ would be relatively high, causing a high number of collisions. Consider for instance the case when the estimated number of remaining nodes is one. Then, τ=1. In this case, if there are at least two nodes trying to transmit, a collision will occur. Hence, the clusters can never be formed, and all nodes would deplete their energy, rendering the network useless. A simple solution to avoid this situation is to establish a probability transmission threshold $\tau_{th}$, in such a way that $\tau <\tau_{th}$. However, the selection of the value of $\tau_{th}$ is not straightforward, as is shown in the Numerical Results section. Indeed, a very low value of $\tau_{th}$ or even a value of $\tau_{th}≃1$ entails higher energy consumption and cluster formation delay due to higher idle listening or collision probabilities.
- In the adaptive transmission probability scheme, three harmful events occur in noisy channels. The first one is similar to the optimal scheme, when the estimated number of active nodes is low, but in fact, there is a higher amount of nodes attempting to transmit. In this case, the estimated value of τ would be relatively high, causing the issues described above. Again, the use of a threshold $\tau_{max}$ is advisable. On the other hand, this adaptive scheme is able to decrease the value of τ if the channel is found idle. The main problem is when a set of consecutive slots has been detected as idle slots, such that τ≃0. In this case, nodes are not able to transmit in subsequent time slots. This case can happen if the false negative probability is rather high. To solve this issue, another threshold, $\tau_{min}$, is proposed to limit the value of τ. Finally, in the adaptive scheme, the value of τ is updated with parameter γ based on the conditions of the channel. Hence, this last parameter has to be carefully selected in order to give a soft change in the value of τ and, thus, avoid collisions and idle listening periods.

## 4. Cluster Head Selection Schemes

In this section, we explain in detail the cluster head selection schemes. When all nodes have successfully transmitted their control packet at the cluster formation phase, the sink node has all the information regarding the nodes that will report information during the steady state phase. This includes the id of such nodes and an estimation of the position of each node.

We consider two intelligent schemes where multiple iterations are performed in order to select the most appropriate nodes to become CHs: fuzzy C-means and K-medoids. Each iteration attempts to reduce energy consumption in the steady state. These schemes are general clustering algorithms that aim at grouping data points that share similar characteristics. These algorithms can be used in wireless sensor networks in order to group nodes to better distribute CH nodes in the supervised area [4]. In this work, we consider the position of the nodes as the characteristic to create the clusters, so that nearby nodes will tend to be included in the same cluster. We also consider a direct clustering scheme, which only requires one iteration to determine the CHs, and we called this K-trans.

Additionally, to evaluate the performance of these algorithms, we consider three different transmission distances from the CH to a cluster member: short distance, medium distance and long distance. Then, to study the performance of these clustering algorithms, we consider that nodes can adapt their power transmission according to the distance to the receiving nodes. As such, energy consumption depends on the principle of the losses by propagation on the free-space described in Equation (23), where *D* is the distance, *f* is the frequency used by the WSN and *c* the speed of light.

(23) $$L_{f}={\left(\frac{4\pi D}{\lambda }\right)}^{2}={\left(\frac{4\pi Df}{c}\right)}^{2}$$

### 4.1. Fuzzy C-Means

Fuzzy C-means uses fuzzy logic to find the optimal centers (CHs in this case). As such, it is possible that CMs belong to more than one cluster according to a membership grade [20]. However, we are considering that CMs are grouped into the CH with the highest value of membership grade. The membership grade is denoted as $u_{i,j}$, which means that the node *i* belongs to the CH *j* with a value $0\leq u_{i,j}\leq 1$, and the sum of all grades of each node must be one.

In Algorithm 1, we depict the fuzzy C-means algorithm. Basically, it finds a solution such that one of both statements is true: no significant progress is made reducing the value of the objective function $J_{m}=\sum_{i=1}^{N}\sum_{j=1}^{C}u_{i,j}^{m}{∥x_{i}-c_{j}∥}^{2}$, or the maximum number of allowed iterations is reached [25].

**Algorithm 1** Fuzzy C-means algorithm.
1:Initialize U, a NxCmatrix with the membership grades2:**for** each iteration *k* **do**3:    Compute the centers set $C\left(k\right)=\left[c_{j}\right]$ using $c_{j}=\frac{\sum_{i=1}^{N}u_{i,j}^{m}x_{i}}{\sum_{i=1}^{N}u_{i,j}^{m}}$4:    Update $U^{\left(k\right)}$ with $u_{ij}=\frac{1}{\sum_{k=1}^{C}{\left(\frac{∥x_{i}-c_{j}∥}{∥x_{i}-c_{k}∥}\right)}^{\frac{2}{m-1}}}$5:    **if** $∥U^{k+1}-U^{k}∥<\epsilon$ **then**6:         Finish the process7:    **end if**8:**end for**


Furthermore, it is important to mention that fuzzy C-means obtains centers that are not necessarily in the set of nodes. This is because each center is a point with the average characteristics of each member, such that it represents the data points belonging to a same cluster. In the literature some variants of the fuzzy C-means algorithm have been presented, which consider centers as part of the dataset, as the well-known Fuzzy clustering with Multi-Medoids algorithm (FMMdd) [26] or the robust version of this fuzzy c-medoids algorithm called Robust Fuzzy c-Medoids Algorithm (RFCMdd) [27]. However, in each iteration, these kinds of algorithms have to compute additional parameters such as the entropy of the dataset, harmonic dissimilarities or sorting processes, which implies extra delay and complexity. Therefore, since the CHs are battery-operated and low-cost nodes and the processing capabilities are restricted, we use the traditional fuzzy C-means algorithm and choose the nodes nearest to each center to act as CHs. Notice that each time that the clusters are formed at the beginning of each round, the fuzzy C-means algorithm finds exactly the same nodes to become CH. This is because the parameter used to form the clusters is the distance among nodes. This would deplete the energy of such nodes faster than the rest of the nodes. Hence, to address this issue, in each new round, we modify this scheme in the following manner. Clusters are formed in the first round where the most suitable nodes to become CHs are selected according to their respective distance to the remaining nodes. Then, in subsequent rounds, the node inside each cluster with the highest level of residual energy becomes CH.

### 4.2. K-Medoids

The the K-medoids algorithm is a version of the K-means algorithm, where centers are selected from the set of data points [19]. This algorithm is shown in Algorithm 2. K-medoids groups the nodes with the lowest distance among them to be part of the same cluster by finding the optimal center such that the nodes associated with the CH are the nearest ones. There are two ways to initialize the algorithm: the first one is selecting the nodes farthest among them such that the clusters are dispersed in the network area, and the second one is by randomly choosing the nodes that will be CH at the beginning of the algorithm. They are called in this paper K-med C/I (which stands for K-medoids cluster-based initialized) and K-med R/I (which stands for K-medoids randomly initialized), respectively. Similar to fuzzy C-means, in each round, K-medoids chooses the same nodes to be CHs, As such, we propose the following modification: In the first round, the algorithm computes the set of CHs with the active nodes. In subsequent rounds, if the set of CHs is different from the previous round, then it maintains the CH set computed; in the other case, the nodes belonging to each cluster are the same, but the CH status is assigned to the node with the highest level of residual energy of each cluster.

**Algorithm 2** K-medoids algorithm.
1:Initialization of the centers (CH)2:Associate each node with its closest CH3:Compute the configuration cost (sum of the distances of each CM to its CH)4:**while** the configuration’s cost decreases **do**5:    **for** each cluster **do**6:        Swap the CH function with a CM node7:        Recompute the new cost8:        **if** the cost increases **then**9:           Undo the swap (keep the previous configuration)10:        **end if**11:    **end for**12:**end while**


### 4.3. K-Trans

The K-trans algorithm is a proposal that randomly selects the CH set. Basically, the CH nodes are selected from the first *K* nodes that transmitted their corresponding control packet. Intuitively, each node has the same probability to transmit, and all nodes have the same probability to successfully transmit their packet. Hence, in principle, CHs should be dispersed along the surveilled area. However, it is not uncommon to find two or more CHs close to each other. This causes CMs to be able to be at a considerable distance from their CHs, causing costly transmissions in the steady state phase. The advantage of such a scheme is that it does not require any processing time at the sink node. Also note that an acting CH can become CH again in subsequent rounds. However, this case is not highly probable if the number of nodes is high. Finally, this algorithm can be used in both a centralized and distributed manner since each node can listen to the control packets of each other active node. Then, each CM is capable of choosing its nearest CH according to the power transmission level detected. This contrasts with the previous algorithms that require the sink node to compute the appropriate CH nodes.

## 5. Numerical Evaluation

In this section, the different transmission probability strategies are numerically studied and compared. Furthermore, we developed a home-written network simulator based on discrete events in C++ in order to compare and validate the results of the mathematical models. In each subsection, we describe the system setup used to obtain the numerical results.

### 5.1. Transmission Probability Strategies

**Figure 6:**
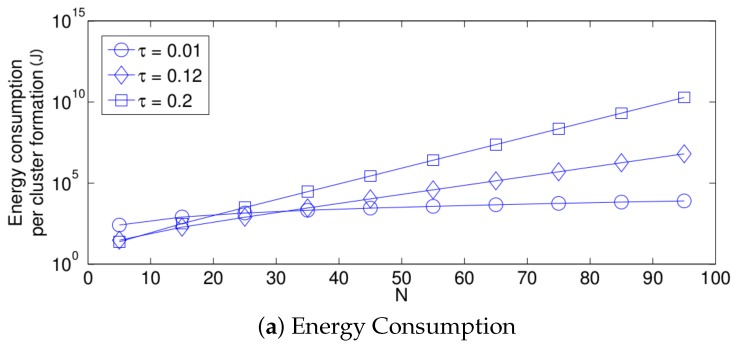

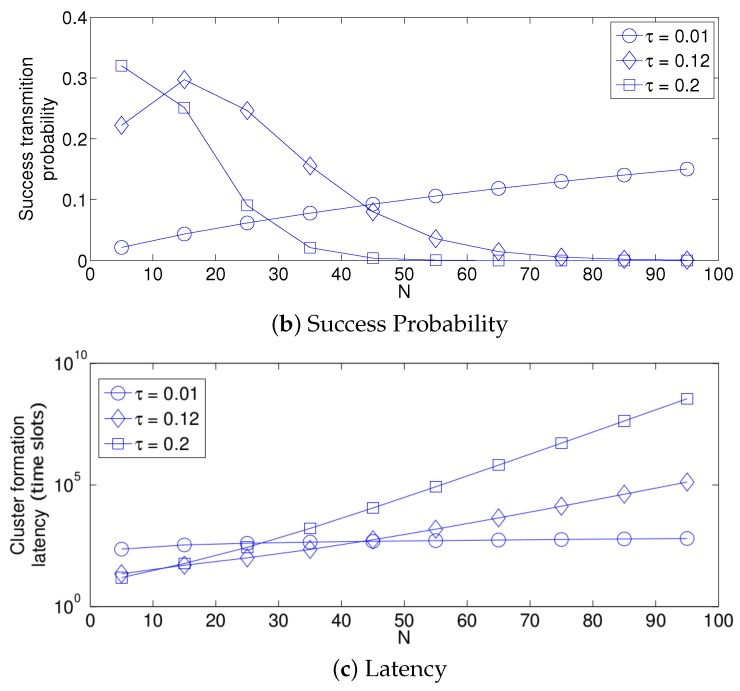
Fixed transmission probability for different values of *N*.

**Figure 7:**
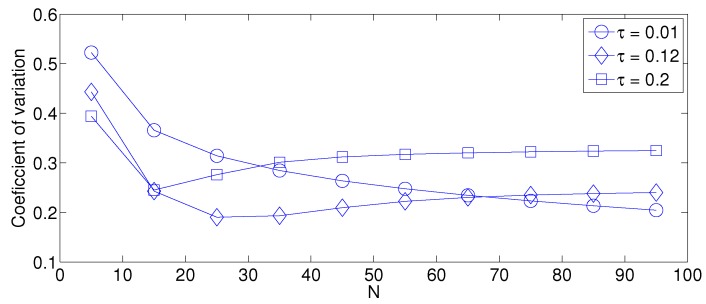
Coefficient of variation for cluster formation latency for different values of *N*.

**Figure 8:**
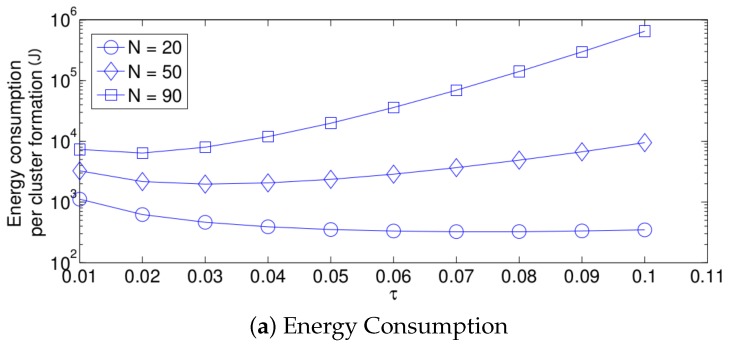

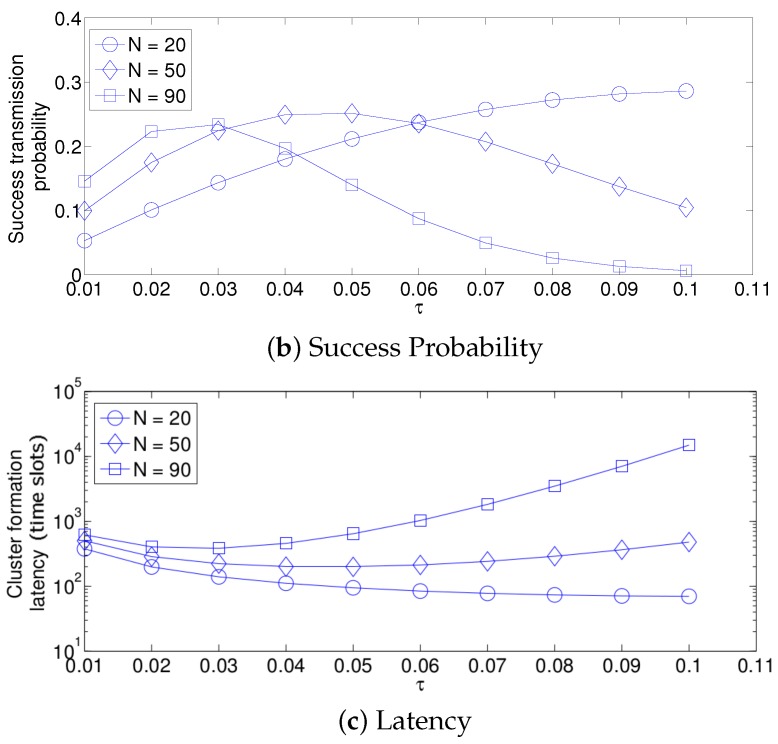
Fixed transmission probability for different values of τ.

First, we focus on the fixed strategy by observing Figure 6, Figure 7 and Figure 8. From the simplified model, it is clear that the energy consumption of the system increases as the network density increases for any value of τ. This is due to the fact that as *N* increases, there are more nodes attempting a transmission. Therefore, there are more transmissions. Furthermore, the cluster formation latency increases for the same reason. On the other hand, the success probability does not have the same behavior. For small values of *N*, the success probability, i.e., the probability that a single packet transmission occurs in a given time slot (in other words, a single packet is transmitted in the network by any node) is small because there are just a few transmissions and there are many idle time slots. It is important to mention that the success transmission probability experienced by each node is high since any packet transmitted by a given node is likely to experience no collision when *N* is low. Also note that from the system perspective, an empty slot is a failure since no packet was successfully transmitted. Building on this, a failure does not necessarily imply a collision. As *N* increases, the success probability increases because there are more transmissions, and now, there are less idle time slots and still not a high number of collisions. However, when the value of *N* is higher than a certain threshold, the success probability begins to decrease. This is because the number of collisions increases, and now, there are just a few single transmissions per time slot. This effect is clearly seen in Figure 6b. Note that for a small value of τ (τ = 0.01), the threshold is beyond the value of N=95, while for a high value of τ (τ = 0.2), the threshold is lower than N=5. For the case of τ=0.12, the threshold is close to N=15. Other interesting observations can be made for this strategy:

- The performance of the system is very sensitive to the value of τ as shown in Figure 6. For low network densities, the value of τ should be high in order to achieve a low energy consumption. For instance, by observing the case where the number of active nodes is relatively small (N=5), a low value of τ (τ=0.01) causes higher energy consumption. This is because the nodes spend a lot of time in reception mode consuming unnecessary energy. On the other hand, for high values of *N*, the transmission probability should be rather small. Observe the case where N=95. A value of τ=0.2 causes a high number of collisions, and consequently, the energy consumption is very high, while a value of τ=0.001 achieves a low energy consumption. Then, it is clear that τ has to be carefully selected depending on the value of *N*. In the case of the values considered in this section, when N<15, the performance of the system is better with τ=0.2. When N=15, the value of τ=0.12 achieves the lowest energy consumption and cluster formation latency, as well as the highest success probability. Conversely, when N>15, the system has the best performance for τ=0.01.
- As mentioned in Section 2, there is a low variation in the cluster formation delay that can be evaluated in terms of the coefficient of variation presented in Figure 7. Since the cluster delay can be calculated by the sum of geometrically-distributed random variables, we can observe that the coefficient of variation is lower than 0.6 for any value of τ and *N*.
- For a practical implementation of the fixed strategy, in order to select an appropriate value of τ, Figure 8 presents the performance of the system for *N* fixed vs. τ. From these results, we can see that for N=90, an appropriate value of the transmission probability is close to 0.02. This corresponds to a case of a very dense network. For a medium-high density network, N=50, a suitable value of τ is 0.04. Additionally, for a medium-low density network, N=20, a suitable value of τ is higher than 0.1.

**Figure 9:**
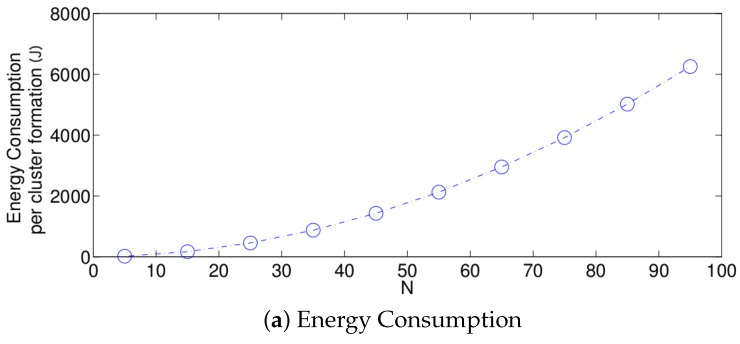

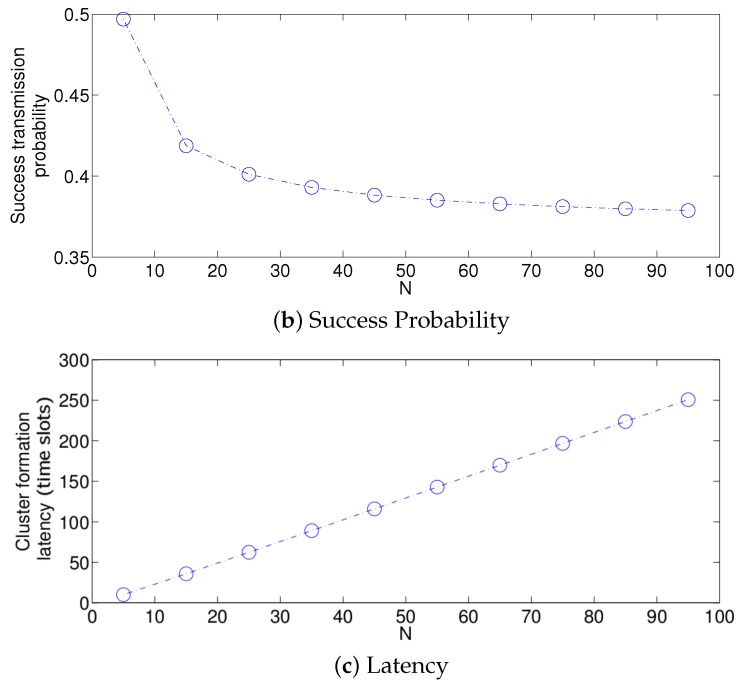
Optimum transmission probability for different values of *N*.

The performance of the optimum strategy is presented in Figure 9. In this scheme, the value of τ that maximizes the success probability is always used. As such, the success probability only decreases as the value of *N* increases and there is no longer a threshold. The reason for this is that, for higher densities, it takes a longer time for the sensor nodes to transmit their control packet successfully due to idle listening and an increasing collision probability.

**Figure 10:**
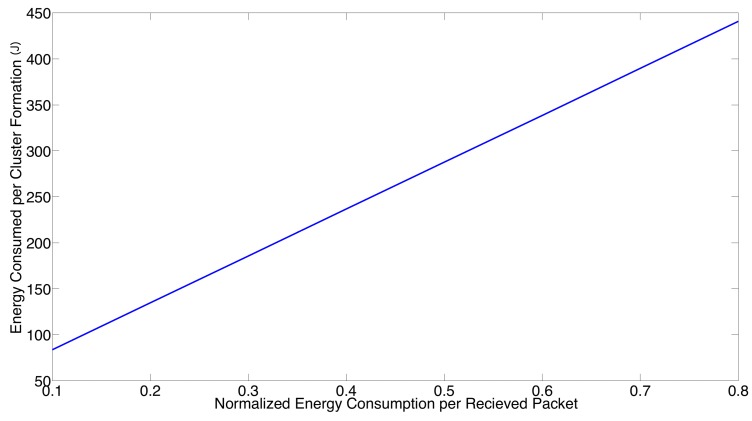
System performance for different values of the normalized energy used per reception of a packet.

Now, the effect regarding the relation between the energy consumed for a packet transmission and reception is studied. Recall that in Section 2, it was considered a normalized energy consumption for a packet transmission, i.e., $E_{t}=1.0$, while the normalized energy consumption for a packet reception was considered to be half of the energy needed for a packet transmission, i.e., $E_{r}=0.5$. Now, we relax such an assumption. In Figure 10, we present the system performance in terms of the energy consumption per cluster formation for different values of $E_{r}$, considering that $E_{t}>E_{r}$. Note that both success transmission probability and cluster formation latency are not affected by the value of $E_{r}$. From this figure, it can be seen that the energy consumption per cluster formation has a linear dependence on the normalized energy consumption per received packet. As such, the numerical results presented in this section with the assumption that $E_{r}=0.5$ can be easily scaled for different values of $E_{r}$.

**Figure 11:**
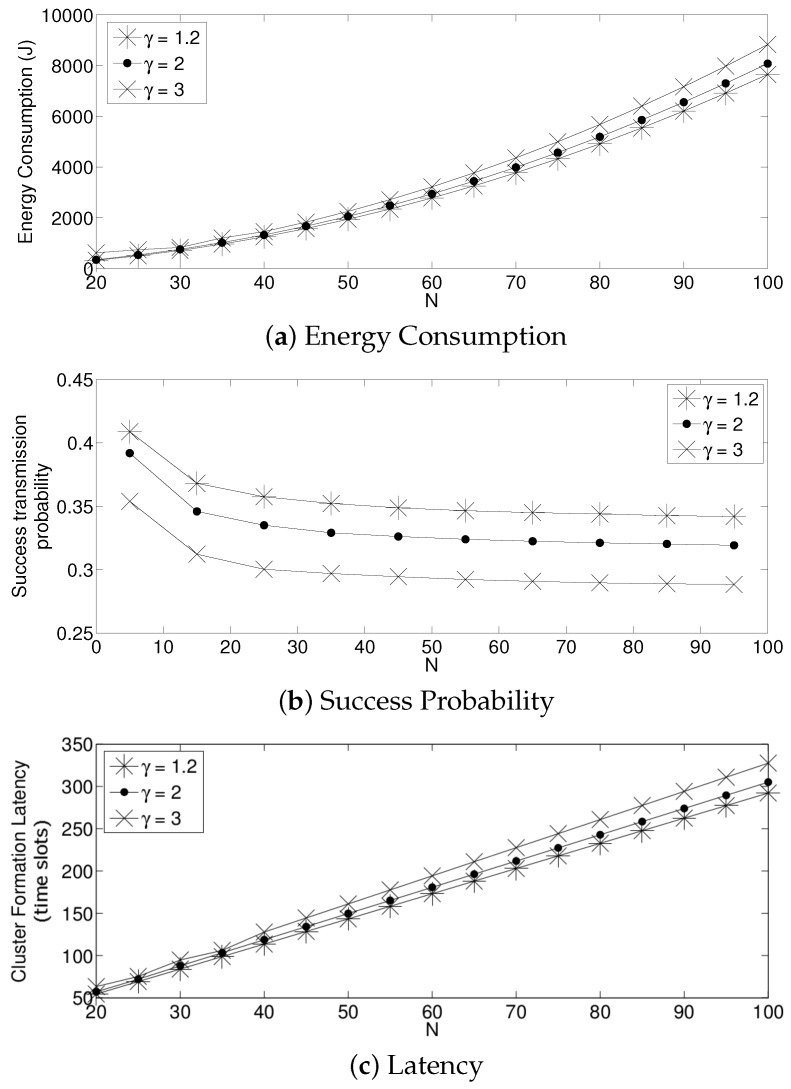
Adaptive transmission probability for different values of *N*.

**Figure 12:**
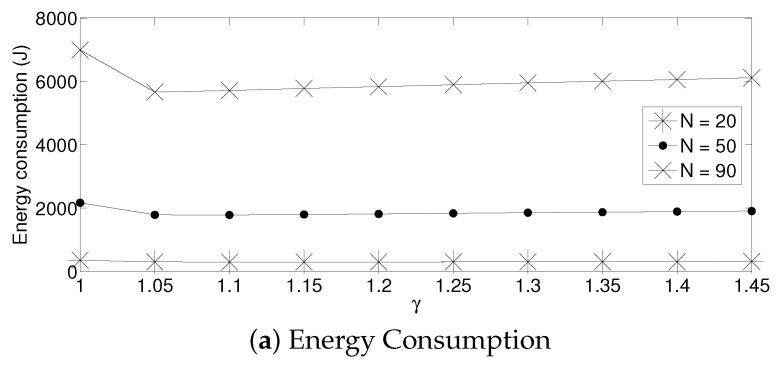

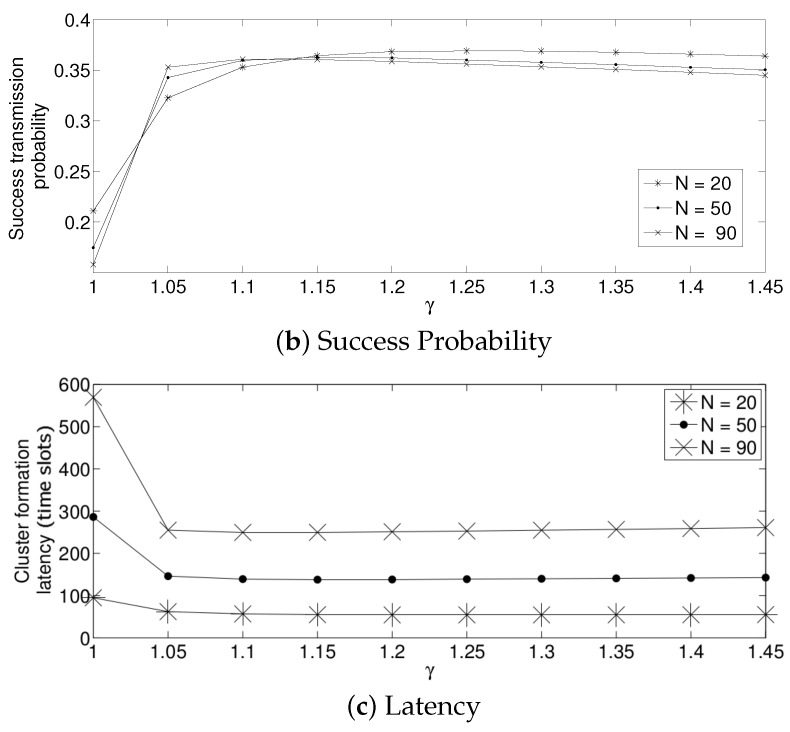
Adaptive transmission probability for different values of γ.

For the adaptive strategy, in Figure 11, it can be seen that the value of γ has to be small for all the values of *N* considered in this paper. In particular, a value of γ≥2 produces a low system performance. The reason for this effect is that if γ is high, the transmission probability suffers a high degree of variation. Indeed, as the nodes sense a collision, each of them decreases the value of τ too much. Therefore, it is very likely that for the next time slot, none of the active sensors will transmit, wasting energy in idle listening mode. Then, after listening to the idle slot, the sensors will increase the transmission probability too much, also augmenting the possibility of having a collision. On the other hand, a small value of γ makes the adjustments on the value of τ in a much smoother manner in the sense that it slightly increments the transmission probability in the case of an idle slot, and it slightly decreases it in the case of collision. Again, for a practical implementation of this scheme, it is important to carefully select the parameter γ. By observing Figure 12, we can see that a suitable value of γ is close to 1.05 for a high density scenario and 1.3 for a medium-low density scenario.

**Figure 13:**
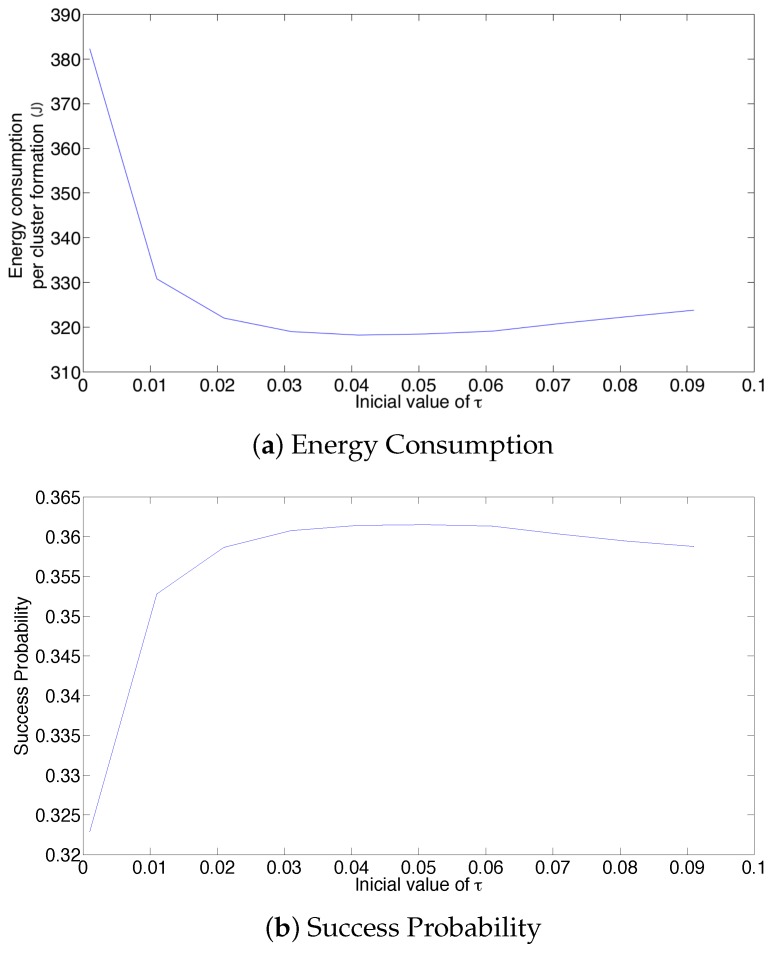

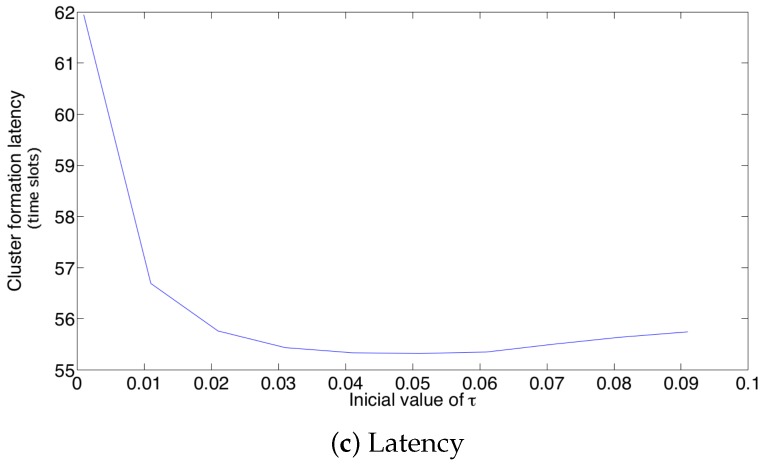
Effect of the initial retransmission probability on the performance of the system, *N* = 20.

Now, the effect of the initial value of the transmission probability is studied. Figure 13 shows the average values of energy consumption, success probability of packet reception and packet latency at the cluster formation phase for 20 active nodes. From these results, it is clear that the initial value of the retransmission probability affects the system’s performance for the adaptive strategy. In particular, a small value on τ renders the lowest success probability and the highest energy consumption and latency. This is because, for such a small value (τ=0.001), the system cannot adjust itself sufficiently fast. As such, there are many empty slots, generating a high energy wastage due to overhearing. It is important to notice that the lowest energy consumption and latency, as well as the highest success probability are obtained for an initial value of τ=0.05, which corresponds to the value of 1/N.

**Figure 14:**
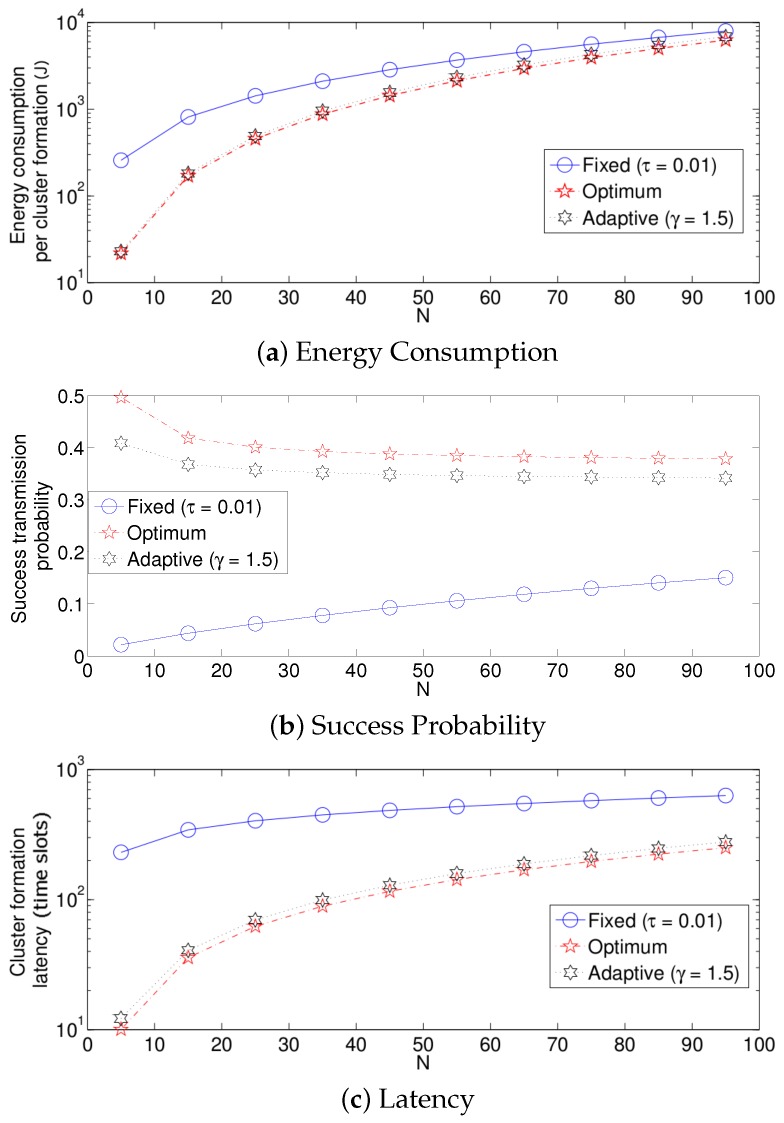
Comparison of transmission strategies.

We now compare the three different strategies considered in this paper. Figure 14 shows the results. For simplicity, in these experiments, we consider τ=0.01 for the fixed retransmission strategy and γ=1.5 for the adaptive scheme. Note that for a practical implementation of a dense WSN, these values render an acceptable performance. Keep in mind that the values of τ and γ have to be adjusted for different network densities. For all three performance parameters, the fixed strategy has the worst results, while the optimum strategy achieves the best results. However, the adaptive mechanism achieves very close results compared to the optimum scheme. The reason for the low performance of the fixed strategy compared to the other two strategies is as follows. Note that in the cluster formation phase, the number of nodes attempting a transmission is constantly decreasing with every successful transmission. Then, for a particular value of *N*, when the cluster formation is beginning, τ should be small since there is a high number of potential transmissions. However, as the cluster formation phase progresses and the number of potential transmissions decreases, the value of τ should be increased. For instance, consider the value of a fixed transmission probability. We can see in Figure 8 that a suitable value for τ is close to 0.02 when N=90. Meanwhile, when many nodes have finished their transmission of the reports to the sink, say for example 20 remaining nodes, a suitable value of τ would be much higher than 0.1. Since in this strategy, the fixed transmission probability is kept constant at 0.1 during the complete cluster formation, this value of τ would cause a higher energy drain due to the idle listening time. On the other hand, if a higher value of τ=0.4 were to be used during the whole cluster formation, the number of collisions at the beginning of this phase would be extremely harmful for the system due to the high collision probability. Another important observation is that the energy consumption for all three schemes presented in this work is similar for high values of *N*. The main reason for this behavior is that the total energy consumption for all three schemes is dominated by the energy consumed when the number of active nodes is large and all three schemes behave similarly at the beginning of the protocol when the number of active nodes is large. The total energy consumption for all three schemes is dominated by the energy consumed when the number of active nodes is large simply because there are more nodes consuming energy. On the other hand, all three schemes behave similarly at the beginning of the protocol because in the case of the fixed scheme, the expected value for the number of slots until a successful transmission is given by the expression $\tau k{\left(1-\tau \right)}^{\left(1-k\right)}$, which is an exponentially-increasing function in *k*, i.e., the optimal fixed probability has to favor large values of the number of active nodes. The same reasoning implies that the fixed scheme behaves badly towards the end of the protocol since it is using a very small transmission probability. This will result in many empty slots. However, this will not make a big difference in terms of energy because the energy consumption during empty slots is much lower than the energy consumption during collisions, strengthening the argument that the total energy consumption for all three schemes is dominated by the energy consumed when the number of active nodes is large.

**Figure 15:**
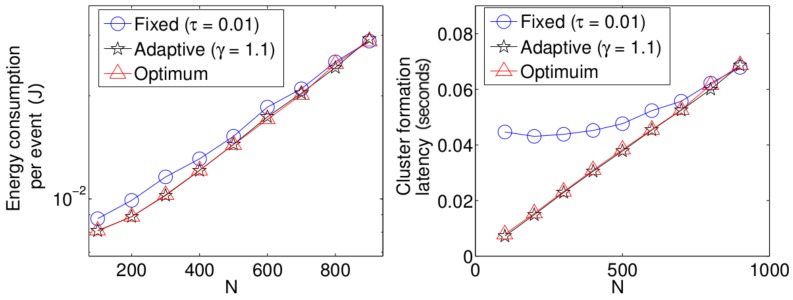
Comparison of simulation results for different transmission strategies.

Now, we present performance results for a more realistic environment. Due to the complexity of the mathematical model, we obtained the following results using the simulation tool developed in C++. In this realistic scenario, nodes can use power control to vary the amount of transmit power. The data packet size *ℓ* (280 bits) is comprised of the data (256 bits), the length of the identification field, *Id* (16 bits), and the *Len* field (8 bits) to specify the length of the payload data. The control packet size is only comprised of the *Id* field. The energy consumed to transmit a packet depends on both the length of the packet *ℓ* and the distance between the transmitter and receiver nodes *d* as is considered in [5]. Namely,

(24) $$\;E_{t}\left(l,d\right)=\left\{\begin{array}{cc} \ell E_{\mathrm{elec}}+\ell \varepsilon_{\mathrm{fs}}d^{2}, & \mathrm{if}\;d<d_{0}\\ \ell E_{\mathrm{elec}}+\ell \varepsilon_{\mathrm{mp}}d^{4}, & \mathrm{if}\;d\geq d_{0} \end{array}\right.$$

where $E_{\mathrm{elec}}$ is the electronics energy, $\varepsilon_{\mathrm{fs}}\times d^{2}$ or $\varepsilon_{\mathrm{mp}}\times d^{4}$ are the amplifier energies that depend on the distance to the receiver and $d_{0}$ is a distance threshold between the transmitter and the receiver over which the multipath fading channel model is used (i.e., $d^{4}$ power loss); otherwise, the free space model (i.e., $d^{2}$ power loss) is considered. The energy consumed at the reception of the packet is calculated according to $E_{r}\left(\ell \right)=\ell E_{\mathrm{elec}}$. For both the simulation model and the analytical model, the network starts with *N* active nodes. Additionally, whenever the number of nodes that have deployed all their energy is over 60 percent of *N*, the network is automatically refilled with new sensor nodes in order to have *N* sensors in the network again. This procedure is repeated 1 ×$10^{6}$ times, and then, the simulation is finished. In Figure 15, we show the average energy consumption and average cluster formation for the studied strategies in such a practical environment. It can be seen that, although different results are obtained, due to the different environment considered, the same conclusions hold that the fixed strategy has the worst performance while the optimal and adaptive schemes achieve the lowest average energy consumption and cluster formation delay.

### 5.2. System Performance in Noisy Channels

In order to observe the impact of the errors in the channel, we present the percentage of increased energy consumption and cluster formation time for networks composed of five nodes to 100 nodes. For these results, we consider that false positive and false negative probabilities are in the range of $0.1\leq P_{e}^{+},P_{e}^{-}\leq 0.5$.

**Figure 16:**
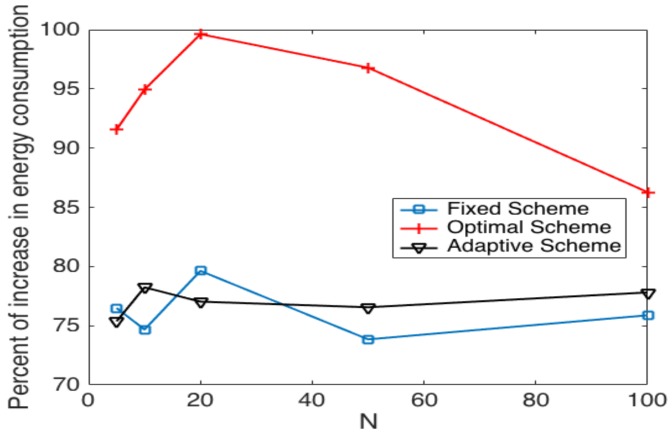
Comparison of the increase in energy consumption in noisy channels among transmission strategies.

Figure 16 shows the average percent of energy consumption increment in the cluster formation process. We can observe that the adaptive scheme has an increase of 75% and 78%, and the highest increase is presented for N=10. The fixed scheme presents an increase that goes from 74% to 80% with the highest increase in N=20. The scheme that presents the highest increase is the optimal, which yields an energy consumption increment from 91.5% to 100%. One important result of this work is that the optimal scheme is more affected by the presence of errors since the value of τ is updated each time a false positive error happens. Adaptive and fixed schemes are less affected by the errors. This is because the fixed probability scheme does not update the value of τ, and the adaptive scheme varies the value of τ relative to the value of γ. Notice that these and the subsequent results were obtained as the average of different evaluation points with error probabilities of $0.1\leq P_{e}^{+},P_{e}^{-}\leq 0.5$. Furthermore, the values of γ considered are in the range of 0.75≤γ≤0.95, with the lowest value for small networks (five nodes), and it increases as the size of the network increases.

**Figure 17:**
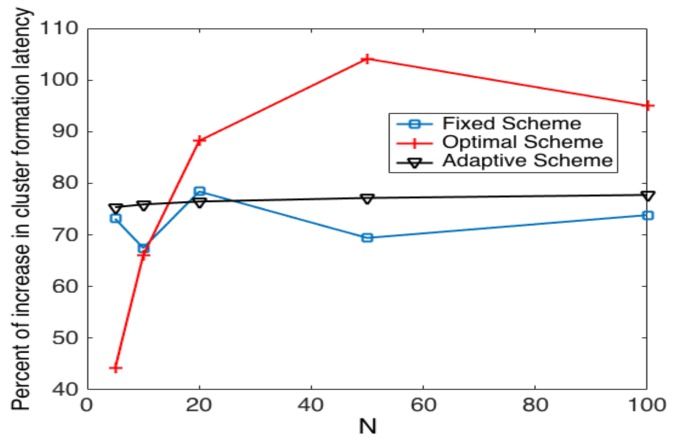
Comparison of the increase in latency in noisy channels among transmission strategies.

On the other hand, the increase of latency is shown in Figure 17, where we can observe that the adaptive strategy has a delay increase from 75% to 78%. The fixed scheme presents an increase from 67% to 78%. However, the optimal strategy presents increases in the range of 44% to 103%. Thus, we can see that the optimal strategy is more vulnerable in the case of errors while the adaptive and fixed schemes remain almost constant for any value of *N*.

**Figure 18:**
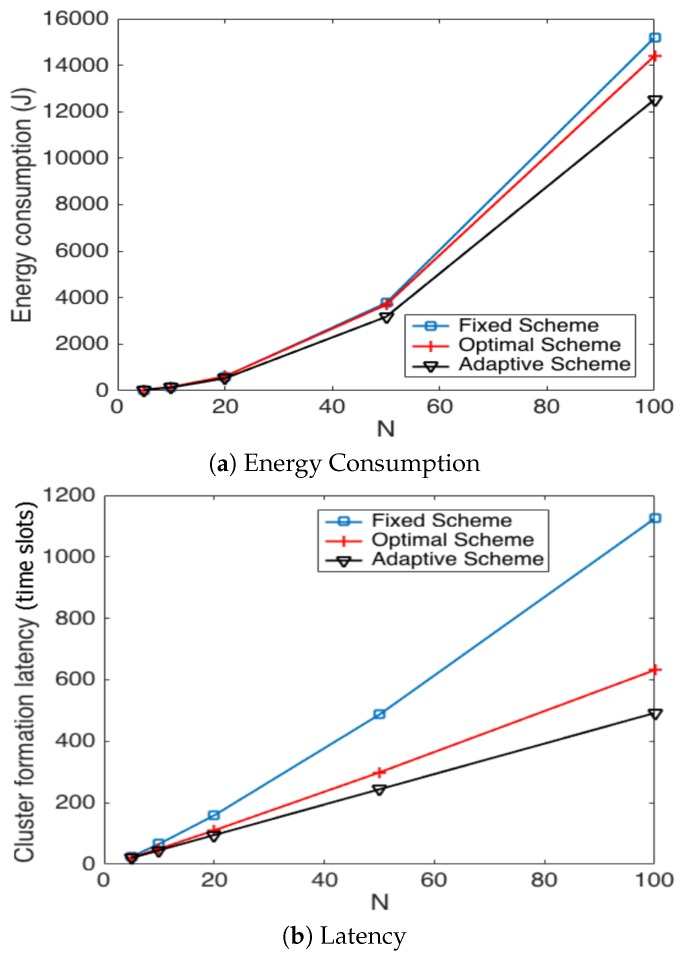
Comparison of transmission strategies in noisy channels.

In Figure 18, we present the average energy consumption and latency during the cluster formation process. It can be seen that both parameters are affected in the presence of errors. However, the best performance in both metrics is achieved by the adaptive scheme. This is because the optimal scheme is more vulnerable to errors in the channel. The fixed strategy always achieves the highest energy consumption and cluster formation delay.

**Figure 19:**
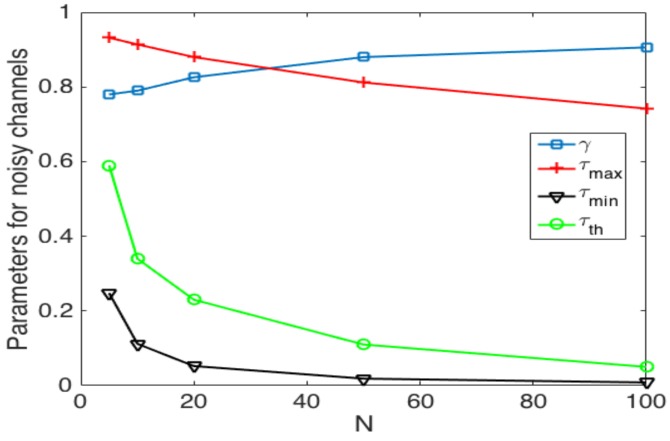
Thresholds of parameters in adaptive and optimal strategies in noisy channels.

Finally, we now study the impact of the selection of the transmission probability thresholds. Figure 19 shows the values of these thresholds and γ that achieve the best performance of the system. We can see that as the number of nodes increases, the value of $\tau_{th}$ decreases. This is because when there are more nodes in the system, in order to avoid collisions, the value of τ has to be kept low. For the case of $\tau_{max}$ in the adaptive scheme, it decreases as the number of nodes increases for the same reason as $\tau_{th}$. On the other hand, the value of $\tau_{min}$ has to decrease as the number of nodes in the network increases and to be constant for N≥50. Finally, for the optimal value of γ, we observe that that has to be in the range of 0.78≤γ≤0.91. As such, the best performance is obtained when the change of value in τ is soft, i.e., no abrupt changes in τ. Clearly, as γ is closer to one, the update of τ is lighter since τ(t+1)=τ(t)γ or τ(t+1)=τ(t)1/γ. The other observation is that $\tau_{th}$ is contained in the space between $\tau_{max}$ and τmin, which means that the optimal strategy entails adequate results using the value of $\tau_{min}<\tau_{th}<\tau_{max}$ in order to avoid the use of the channel for more than one node, effectively avoiding collisions.

These results give very detailed guidelines for the selection of the random access protocol in both noisy and error-free channels.

### 5.3. Cluster Head Selection Schemes

For the cluster selection evaluation, the following parameters are considered: *N* total nodes in the system, surveilled squared area of *D* meters per side, *k* CHs, large range transmission of $d_{l}>\;50$ m, medium-range transmission of 25 (m) $<d_{m}\leq 50$ (m) and short-range transmission of 25 (m) $\leq d_{s}$. The default values for these experiments are: D=100 (m); the energy consumed by a long-range transmission is $E_{d_{l}}=1$ unit (50 nJ/bit); medium-range transmission consumes $E_{d_{m}}\;=\;\frac{1}{9}$ units (5.5 nJ/bit); and short-range transmission consumes $E_{d_{s}}=\frac{1}{36}$ units (0.138 nJ/bit). In these results, the energy consumption depends on the transmission range in the steady state (in the cluster formation phase, nodes transmit with the highest transmission power to reach the sink), i.e., we assume that nodes are able to adapt their transmission power according to the distance to the receiver node. Note that if nodes have a single transmission power level in the steady state, there would be no difference between the different cluster selection strategies. Indeed, if nodes consume the same energy level regardless of the position of the CHs, a good CH distribution would not impact the performance of the system. We also focus on scenarios where no singleton clusters are formed (clusters composed only by a CH and no CMs). This is because singleton clusters consume much more energy, and the effect of the distribution of the CHs is no longer relevant. To this end, we consider that k<N/2.

**Figure 20:**
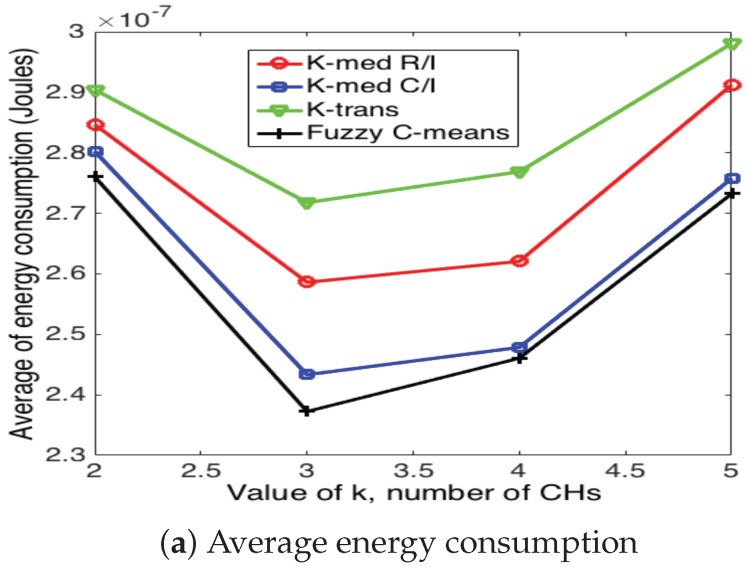

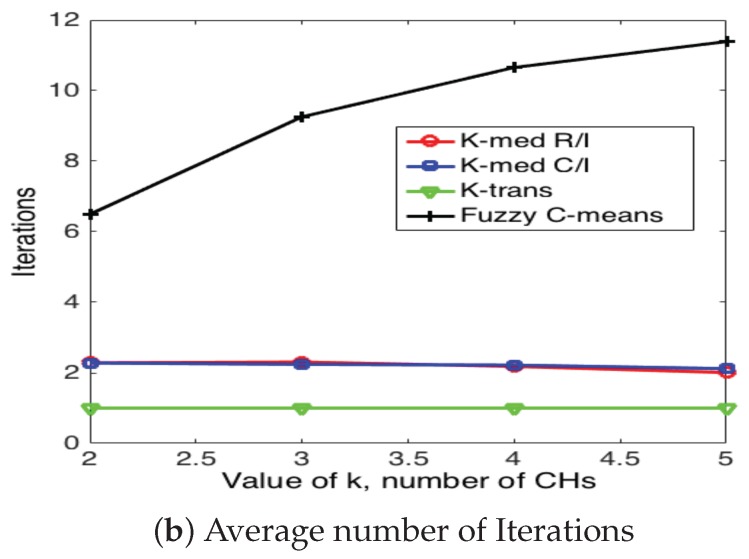
Clustering results for 10 nodes.

**Figure 21:**
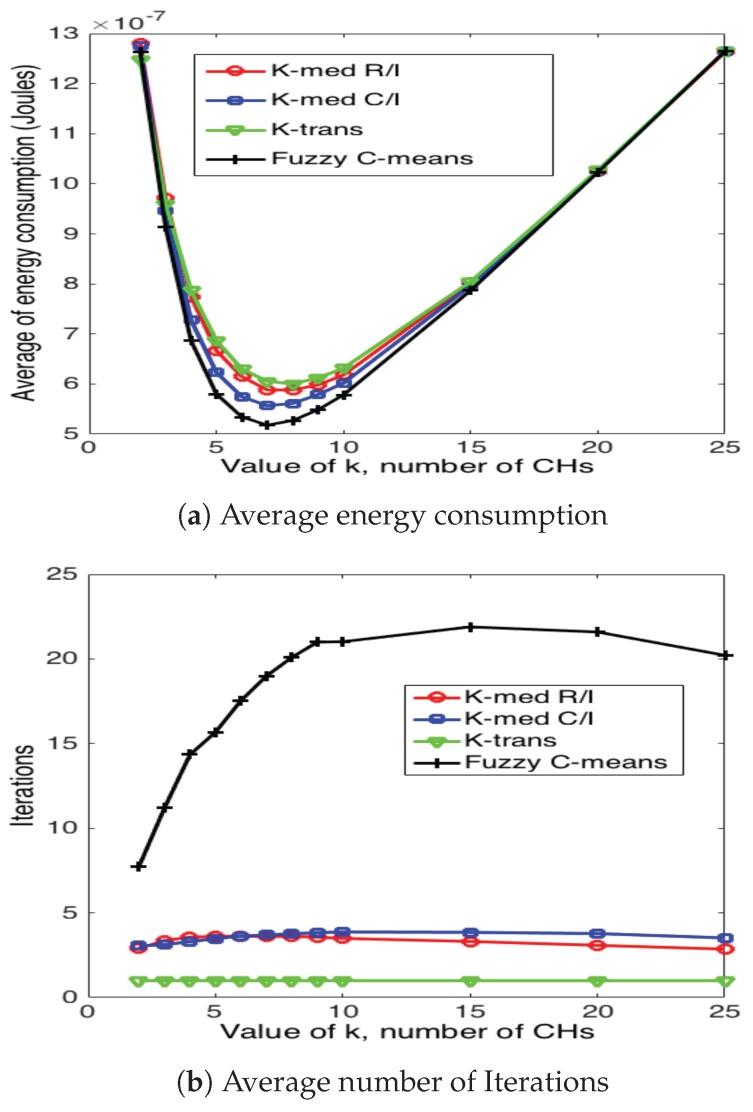
Clustering results for 50 nodes.

**Figure 22:**
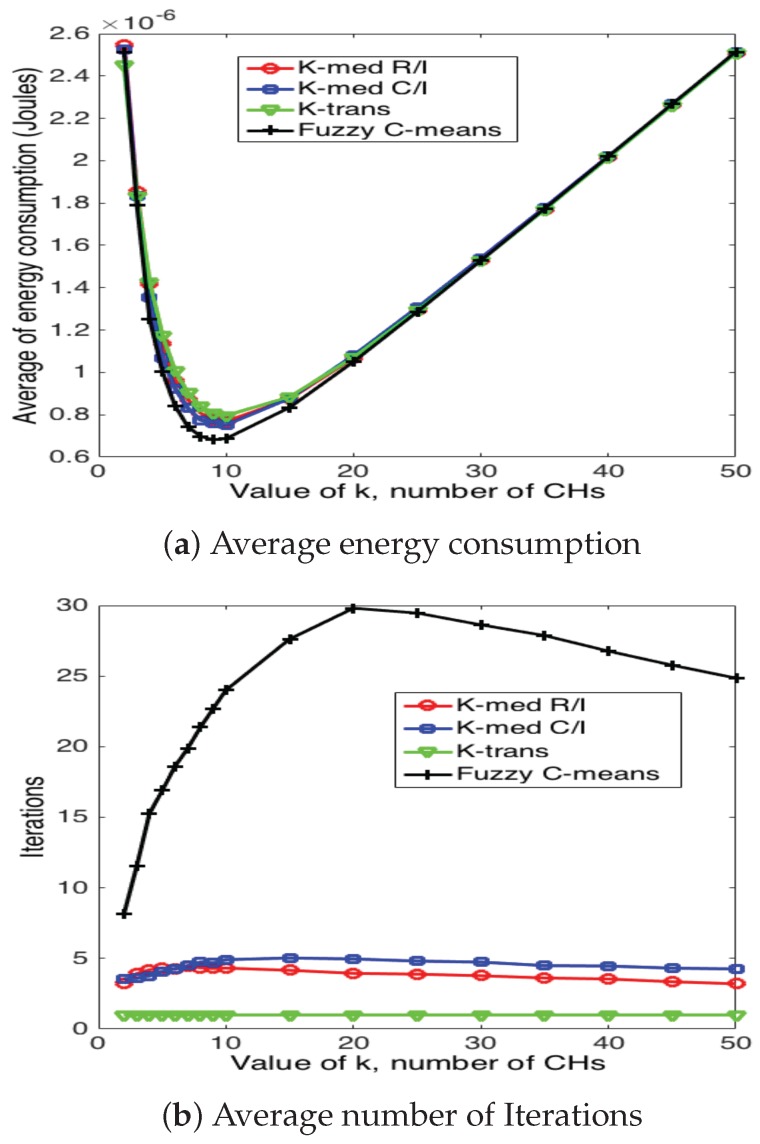
Clustering results for 100 nodes.

Figure 20, Figure 21 and Figure 22 present the average energy consumption and average number of iterations for 10, 50 and 100 nodes in the network, respectively. It can be seen that the scheme that consumes the lowest energy levels is the fuzzy C-means algorithm for any number of nodes. This implies that this scheme selects the CH nodes in the most efficient manner in such a way as to reduce the distance between CH and their corresponding CMs. On the other hand, the fuzzy C-means algorithm is also the one that requires the highest number of iterations for any number of nodes and number of CHs. Note that the average energy consumption between fuzzy C-means is not considerably higher than K-medoids (for any variation), while the number of iterations is considerably higher between these two schemes. For instance, consider the case of N=50 and k=7; fuzzy C-means consumes 10.3% less energy than K-medoids, while it requires 350% more iterations. Recall that while the clusters are forming, the WSN is not reporting data to the sink node. Hence, this time is of paramount importance for the performance of the system. Building on this, the k-trans scheme is always the most energy consuming, but only requires one iteration. Furthermore, the energy consumption of this scheme is not much higher than the intelligent schemes. For instance, when N=100 and k=10, the k-trans scheme consumes 18.3% more energy than the fuzzy C-means and 7.33% more than the k-medoids schemes.

Another important observation is that the number of clusters not only depends on the number of nodes, but also on the particular scheme. For instance, when N=50, fuzzy C-means achieves the lowest energy consumption when k=7, while the rest of the schemes achieve its minimum at k=8.

## 6. Conclusions

This work focuses on the study of three schemes for selecting transmission probabilities for cluster-based WSNs and the impact of different algorithms for selecting CHs in clustered-based WSNs. The resulting WSN has been modeled, analyzed, simulated and studied. The system is analyzed in terms of the energy consumption, success probability and cluster formation latency. From the results derived in this work, it can be seen that a fixed transmission probability is easy to implement in a practical system. However, it has the worst performance. The optimum strategy achieves the best results, as expected, but its implementation is not feasible. The use of an adaptive transmission strategy achieves a performance close to the optimum scheme, and its practical implementation is feasible. As an additional feature of this work, we present several practical guidelines for the selection of the different parameters of each strategy, including an approximation of the optimal scheme, which considers the average number of nodes inside the event area. However, this approximation degrades the performance of the network. Finally, the adaptive strategy produces the best system performance, and at the same time, it allows a simple implementation in a practical system.

As an important contribution of this work, the impact of experiencing a noisy channel over the different strategies was studied, analyzed and mathematically modeled. Clearly, the optimal strategy is the most affected by an error-prone medium, as it is extremely vulnerable to errors while the adaptive scheme is the most robust strategy in the presence of errors. Furthermore, very detailed guidelines are provided to adequately select the different parameters of the system such as the probability transmission thresholds and the value of γ with and without errors.

Additionally, the use of intelligent clustering algorithms for the appropriate CH selection has been analyzed in terms of average energy consumption and the delay added by the algorithms. From the results obtained in this work, it can be observed that the use of iterative clustering algorithms entails a better performance in terms of energy consumption over random selection. However, the price to pay is the necessity of the centralized process that demands higher computer capabilities and implies a higher number of iterations, increasing the time that clusters are formed and, consequently, increasing the time that the network is not reporting to the sink. It is important to note that the added delay of the intelligent schemes was evaluated in terms of the number of iterations to choose the adequate cluster heads. Depending on the computer capabilities of the sink node, this added delay can be negligible since these algorithms are polynomial and are performed in a centralized manner, i.e., it is computed in the same sink node with no need to transmit extra packets. To summarize, if the sink node has sufficient hardware capabilities (in terms of memory and computation power), the use of the intelligent algorithms reduces the average energy consumption in the steady state with little added delay.

As future research works, we are considering different approaches for the selection of the transmission probability such as the use of game theory or machine learning to better select this value in both noisy and dynamic systems where nodes enter and leave (due to mobility, or failure, or energy depletion) the monitored area. Furthermore, another course of research is focused on applying the proposed transmission schemes to more complex systems, such as cognitive radio systems where secondary nodes have to choose an adequate transmission probability to opportunistically select the proper channel without interfering nodes in the primary system.
